# Adaptation of Enhanced Recovery After Surgery Protocol for Elective Gastrointestinal and Hepatopancreaticobiliary Surgeries for Tertiary Hospitals in Ethiopia: A Modified Delphi Study

**DOI:** 10.1002/wjs.70274

**Published:** 2026-03-02

**Authors:** Wongel Tena Shale, Abraham Teshome Sahilemariam, Tilahun Habte Nureta, Tadesse Girma Moges, Edosa Kejela Keno, Wondu Reta Demissie, Robert K. Parker, Mercedes Pilkington

**Affiliations:** ^1^ Department of Surgery Jimma University Jimma Ethiopia; ^2^ Department of Anesthesiology, Critical Care, and Pain Medicine Jimma University Jimma Ethiopia; ^3^ Medical Faculty Institute of Health Jimma University Jimma Ethiopia; ^4^ Department of Surgery AGC Tenwek Hospital Bomet Kenya; ^5^ Department of Surgery Brown University Providence Rhode Island USA; ^6^ Department of Surgery University of Toronto Toronto Ontario Canada

**Keywords:** enhanced recovery after surgery, ERAS, Ethiopia, gastrointestinal surgery, hepatopancreaticobiliary surgery, LMIC

## Abstract

**Background:**

Patients in low‐ and middle‐income countries (LMICs) lack access to safe and affordable surgical and anesthetic care. Standardized evidence‐based perioperative care recommendations and instruments to assess guideline compliance and results are needed. Enhanced recovery after surgery (ERAS) protocols are already in use and have evidence supporting its efficacy. Although ERAS programs have been beneficial in various fields of surgery, they are not widely used in developing countries compared to developed countries. Difference in dietary patterns, living standards, healthcare innovations, and sociodemographic indexes may preclude the direct adoption of existing protocols. The aim of this study is to adapt the ERAS protocol for gastrointestinal (GI) and hepatopancreaticobiliary (HPB) elective surgeries in a way that is feasible, sustainable, and effective for Ethiopian tertiary hospitals.

**Methods:**

A modified Delphi process was used to devise an ERAS protocol for perioperative care of patients who undergo elective gastrointestinal and hepatopancreaticobiliary surgery from preexisting guidelines to fit the Ethiopian context. Thirty‐two panelists were invited from target disciplines to participate in the Delphi after being sampled using purposive and snowballing sampling techniques. Two rounds were conducted until a consensus of 80% was reached on different components of the protocol. Data are presented in aggregate after deidentification.

**Results:**

Thirty‐two experts completed round one and 24/32 completed round two after which mature consensus was achieved. There are eight preoperative recommendations, seven intraoperative recommendations, and six postoperative recommendations that have been adapted from existing guidelines and six novel components. These guidelines were deemed appropriate for 7 types of surgical procedures.

**Conclusions:**

This adapted protocol consisting of 27 recommendations represents a critical step toward implementing standardized resource‐appropriate ERAS pathways for GI and HPB surgeries in Ethiopia.

## 1 Introduction

According to the Lancet Commission and Global Surgery, patients in LMICs are more likely to experience complications and die [1, 2]. LMICs account for 96% of all perioperative deaths globally, and these deaths have a startling economic impact—2.6% of LMICs' total gross domestic product [2, 3]. Despite the widespread perception that delayed presentation and limited access to care are the main causes of unfavorable outcomes in LMICs, inadequacies in the quality of accessible care constitute a significant concern [3]. Standardized evidence‐based perioperative care recommendations and tools to assess guideline compliance and results are clearly needed [2].

Enhanced recovery after surgery (ERAS) is a multidisciplinary approach to perioperative care. A preoperative, intraoperative, and postoperative care protocol is implemented with the goal of improving patient recovery, facilitating earlier discharge, and potentially lowering healthcare costs without increasing complications or hospital readmissions [4]. A recent meta‐analysis of 74 studies demonstrated that ERAS programs significantly reduced hospital length of stay, with a pooled mean decrease of 1.88 days overall and 2.83 days for postoperative stay compared with conventional care. ERAS was also associated with a 29% reduction in overall postoperative complications (RR 0.71 and 95% CI 0.59–0.87) and a similar reduction in 30‐day complications (RR 0.73 and 95% CI 0.56–0.94) [4].

Although ERAS programs benefit many surgical fields, they are less widely applied in LMICs compared to HICs [5]. There is a dearth of evidence regarding the practice of enhanced recovery after surgery in African countries [6]. Differences in diet, living standards, healthcare systems, and sociodemographic limit direct adoption of Western protocols, and tailored perioperative guidelines for GI and HPB surgery remain lacking in Ethiopia. A systematic review emphasized adapting ERAS to local contexts for successful ERAS implementation [2, 7]. To address these gaps, our study aims to adapt ERAS protocols to the Ethiopian context through a modified Delphi consensus, enabling expert‐driven locally feasible solutions.

## 2 Methods

The core protocol development (CPD) team consisted of different disciplines of surgery and anesthesia care. The team was composed of eight members, including four general surgeons, a general and gastrointestinal oncology surgeon, a pediatric surgeon with experience in adapting ERAS protocols and leading implementation efforts, an anesthesiologist, and a PhD student focusing on nutrition. Six of these experts were Ethiopian. A Delphi panel consensus was utilized to create an adaptation of ERAS protocol for patients who undergo elective GI and HPB surgeries in tertiary hospitals in a resource limited set up such as Ethiopia. The Delphi process was evaluated based on established methodological principles, including criteria for panel selection, anonymity, controlled feedback, iteration, and consensus [8]. This work is reported in accordance with the CREDES guideline in conducting and reporting Delphi studies [9].

### 2.1 Identification of Problem Area

Two authors carried out a thorough targeted scoping review. The CPD engaged in open‐ended discussions to identify key problem areas with relevant stakeholders. CPD team referred to the most recent ERAS Society guidelines for perioperative care in elective colorectal surgery as well as the guidelines for abdominal and pelvic surgeries in LMICs, to determine the levels of evidence and recommendation grades for the various components [1, 2, 3, 10].

### 2.2 Expert Panel Selection

We defined experts as general surgeons, hepatopancreatic‐biliary surgeons, colorectal surgeons, gastrointestinal oncology surgeons, cardiothoracic surgeons, pediatric surgeons, anesthesiologists, and surgical fellows who have experience with, or interest in, implementing ERAS protocols within their respective specialties and subspecialties. The panel was heterogeneous consisting experts from different disciplines and geographical locations (predominantly from Ethiopia but including experts from other LMICs and HICs). Thirty‐two panelists were invited to participate in the Delphi. Panelists were invited and recruited until all the targeted health professional disciplines were included. Purposive sampling and snowballing were employed to enroll experts into the panel.

### 2.3 Consensus Technique

Reaching predefined consensus between panelists was used as one of the closing criteria. A priori was set at 80% for consensus. Stability of consensus, defined as the consistency of responses between successive rounds of the study, was also used as closing criteria. A virtual meeting was scheduled to address unresolved issues, but the second round resulted in near‐total agreement, making the meeting and any further rounds unnecessary. Data were presented in aggregates. Percentage was used to identify the rate of group agreement.

#### 2.3.1 Round One

In Round one, an online survey was used to generate opinions on:

1. Scope of protocol
2. Components of intervention to be included and excluded based on previous ERAS guidelines [1, 3, 10, 11].
3. Additional novel recommendations

Five‐point Likert scale was used to decide inclusion and 80% consensus was set as a priori for a recommendation to be considered for the 2nd round Delphi. Open‐ended questions were used to generate novel recommendations. In the first round, we asked panelists to provide specific recommendations on the proposed elements and to suggest any new items that might be relevant in low‐income settings but are not included in existing ERAS guidelines. Suggested novel elements could relate to any of the 3 perioperative phases or extend beyond them. Feedback on established recommendations was reviewed by the CPD team and incorporated when aligned with current evidence. The new suggestions were synthesized into eight candidate elements and returned to the panel for voting on their inclusion in the final protocol using the same five‐point Likert scale.

#### 2.3.2 Round Two

After the first round, the data obtained were analyzed and presented back to all the experts. All free‐form comments were incorporated as part of the feedback, when deemed necessary by the CPD. Experts were encouraged to revise their earlier answers considering round one. Specific recommendations were put forward, and agreement on their inclusion was reassessed using a 4‐point Likert scale: 1—include without changes, 2—include with modifications, 3—include in a different section of the protocol, and 4—exclude from the protocol. Panelists were given open‐ended questions to propose changes to the listed items, either by modifying existing recommendations or suggesting new items particularly relevant to a tertiary hospital in a low‐income country such as Ethiopia. The novel items proposed in round one were similarly assessed for inclusion in the adapted protocol.

The CPD team sought input from professionals with expertise in nursing and pharmacy who specialize in postoperative pain management as well as from nutrition and dietetics specialists with experience in a tertiary teaching hospital in Ethiopia. Although they were not included in the expert panel, since their areas of expertise did not allow them to respond to all or most sections of the survey, they were specifically asked to contribute to the sections of the adapted protocol relevant to their respective domains, and the final protocol was refined accordingly. Prior to implementation, additional stakeholders, including patients, hospital clinical, and academic leaders, and the Federal Ministry of Health Innovation and Quality Office were also consulted. Although their feedback did not inform the content of the protocol itself, it guided the development of the implementation strategies.

### 2.4 Data Collection Period

Data were collected starting August 15, 2024, until saturation was attained by February 28, 2025.

## 3 Results

### 3.1 Panel Participation and Demographics

Thirty‐two panelists were invited and all participated in the first round, achieving a 100% response rate. The panel included general surgeons (*n* = 10), colorectal surgeons [6], anesthesiologists (*n* = 5), HPB surgeons [5], a cardiothoracic surgeon, a pediatric surgeon, a GI surgeon, and surgical fellows [3]. Participants had between 1.5 and 31 years of professional experience, with a mean of 9.02 years. The countries of current or past practice among panelists included Ethiopia, Kenya, Rwanda, Canada, Sweden, India, Pakistan, Nigeria, and the United States. At the time of the study, 83% of participants were actively practicing in Ethiopia. In the second round of these Delphi process, 24 of the 32 prior panelists responded (75% response rate; see Table 1).

**TABLE 1:** Panel participation and demographics.

Category		Count	Percentage (%)
Profession	General surgeon	10	31.25
	Colorectal surgeons	6	18.75
	Colorectal surgery fellows	2	6.25
	Anesthesiologist and critical care specialist	5	15.625
	HPB surgeon	5	15.625
	HPB surgery fellow	1	3.125
	Pediatric surgeon	1	3.125
	GI‐oncology surgeon	1	3.125
	Cardiothoracic surgeon	1	3.125
Country served/worked	Ethiopia	26	81.25
	Canada	1	3.125
	Kenya	1	3.125
	United States	1	3.125
	Rwanda	2	6.25
	USA	1	3.125
	Pakistan	1	3.125
	Sweden	2	6.25
	India	2	6.25
	Taiwan	1	3.125
	China	1	3.125
	Norway	1	3.125
	Somalia	1	3.125
	Nigeria	1	3.125
Years of experience in the field	< 5 years	8	25
	5–10 years	16	50
	11–20 years	2	6.25
	> 20 years	6	18.75

Abbreviations: HPB: hepatopancreaticobiliary, GI: gastrointestinal.

More than half of the participants (53.3%) reported that no formal ERAS program existed in their respective institutions, whereas 40% indicated that some elements of ERAS were implemented informally. However, there was inconsistency in these responses within institutions. Although some surgical respondents believed informal ERAS practices were in place, anesthesiologists from the same institutions often reported the absence of such programs.

### 3.2 Phase 1 Delphi Process Result

During the first round, panelists were asked to evaluate 31 proposed ERAS intervention elements for inclusion based on their relevance and feasibility within Ethiopian tertiary hospital settings (see survey Supporting Information S1: Appendix 1). The expert panel demonstrated strong consensus on a comprehensive range of ERAS components across the preoperative, intraoperative, and postoperative phases, emphasizing key elements that form the foundation of enhanced recovery protocols (see Table 2).

**TABLE 2:** Round one results assessing potential items to include within the scope of perioperative pathway for elective GI and HPB surgery patients at tertiary hospitals in Ethiopia.

Components		Must exclude	Should exclude	Neutral	Should include	Must include	Agreement (%)
Preoperative	Preadmission information, education, and counseling	—	—	—	5	27	100
	Assessment of functional status or frailty	—	—	—	3	29	100
	Optimization (smoking and alcohol cessation, HIV screening, and anemia screening)	—	—	—	9	23	100
	Preoperative nutritional care	—	—	—	12	20	100
	Preoperative fluid and electrolyte therapy	1	1	1	11	18	90.3
	Selective mechanical bowel preparation	—	—	4	16	12	87.5
	Preoperative fasting and carbohydrate loading	—	—	3	12	17	90.3
	PONV prophylaxis	—	2	4	13	13	81.25
	Premedication (selective anxiolytic)	—	—	9	15	8	71.9
	Hair removal	2	5	10	8	7	46.8
	Preoperative biliary drainage in cholestatic liver	4	2	14	8	4	37.5
	Preoperative administration of steroids for planned liver resection	2	3	16	6	4	31.25
Intraoperative	Surgical safety checklist	—	—	—	2	30	100
	Antimicrobial prophylaxis	—	—	—	2	30	100
	Venous thromboembolism (VTE) prophylaxis: Compression stocking and/or intermittent pneumatic compression together with either a LMWH or unfractionated heparin	—	—	1	16	15	96.9
	Multimodal opioid sparing analgesia: Short‐acting opioid sparing analgesia combined with local and regional blocks. TEA, TAP/RA/Subcostal blocks, epidural catheter, and wound catheter.	—	—	1	16	15	96.9
	Standard anesthesia protocol: Short‐acting anesthetic agents, lung‐protective ventilation, and complete reversal of neuromuscular blockade	—	—	1	9	22	96.7
	Fluid balance: Near‐zero fluid balance. Goal‐directed fluid therapy (GDFT).	—	—	2	12	18	93.8
	Normothermia: Active versus passive warming	—	—	4	15	13	87.5
	Surgical access: Open versus MIS: Different types of incisions for open surgery	1	—	8	13	10	71.8
	Drains (peritoneal cavity and pelvis)	—	4	9	13	6	59.4
	Nasogastric tubes (NGT)	2	3	9	12	6	56.3
Postoperative	Postoperative fluid and electrolyte therapy	—	—	—	10	12	100
	Postoperative nutritional care	—	—	—	6	26	100
	Postoperative analgesia	—	—	—	4	28	100
	Early structured mobilization plan			1	3	28	96.7
	Standardized criteria for discharge	—	—	1	10	21	96.7
	Plan for opioid minimization	—	—	1	10	21	96.7
	Prevention of postoperative ileus	—	—	2	12	18	93.7
	Postoperative glycemic control and intensive insulin treatment in the ICU	—	—	8	12	12	75
	Urinary drainage	3	4	8	9	9	56.25

*Note:* The grey shade sets apart the items that did not meet the consensus criteria, which is 80% agreement.

Abbreviations: GDFT: goal directed fluid therapy, ICU: intensive care unit, MIS: minimally invasive surgery, NGT: nasogastric tube, PONV: postoperative nausea and vomiting, RA: regional analgesia, TAP: transverse abdominis plane, TEA: thoracic epidural analgesia, VTE: venous thromboembolism.

Expert panelists suggested several novel and context‐specific interventions deemed important for inclusion in the ERAS protocol (see Table 3).

**TABLE 3:** Suggested context relevant novel elements and rationale for inclusion.

Assessment of social support and social determinants of health	Experts emphasized the need to incorporate a structured assessment of patients' social circumstances and support systems, recognizing their significant influence on surgical outcomes and recovery.
Preoperative preparation of patients with obstructive jaundice (OJ) patients	A focused preoperative optimization protocol for patients with obstructive jaundice was proposed, including fluid and electrolyte correction, administration of vitamin K, and appropriate antibiotic prophylaxis.
Preoperative respiratory exercises	Routine implementation of respiratory exercises preoperatively was recommended to reduce pulmonary complications and enhance postoperative recovery, especially in high‐risk patients.
Establishment of a hospital ERAS team	The formation of a dedicated multidisciplinary ERAS team within each hospital was identified as a foundational requirement for successful implementation and adherence to protocol elements.
Transfusion guidelines and strategies to minimize blood loss	The development of context‐specific transfusion guidelines, alongside intraoperative and perioperative blood conservation strategies, was highlighted as a critical component of enhanced recovery efforts.
Hospital‐to‐home transition planning with a case manager	The inclusion of a designated case manager to oversee the hospital‐to‐home transition process was recommended to support continuity of care, early discharge planning, and improved postoperative follow‐up.
Continuous medical education (CME) in ERAS	Panelists stressed the importance of sustained CME initiatives to improve clinician familiarity with ERAS principles and to encourage protocol adoption and adaptation.
Protocol audit and feedback mechanism	Regular audit and feedback were proposed to ensure quality control, monitor adherence, and identify areas for continuous improvement within the ERAS framework.

Abbreviations: CME: continuing medical education, ERAS: enhanced recovery after surgery, OJ: obstructive jaundice.

### 3.3 Phase 2 Delphi Process Result

**FIGURE 1:**
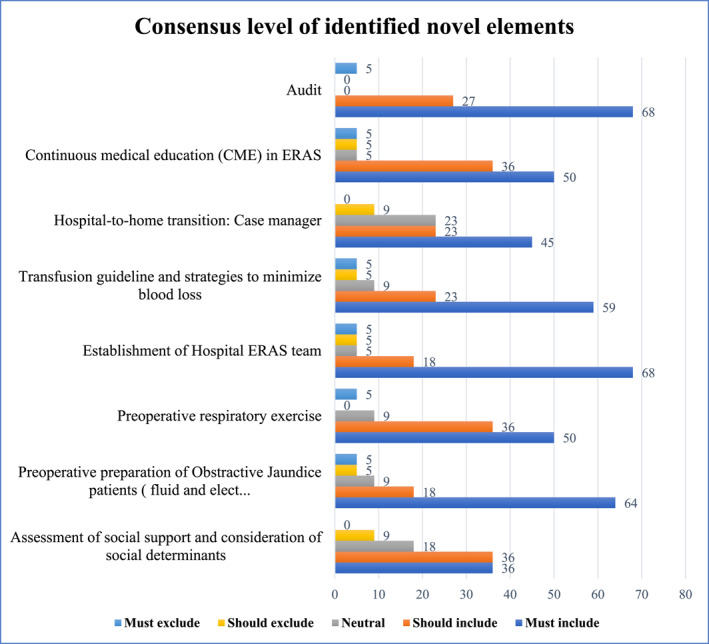
Consensus level of identified novel elements for inclusion.

In the second phase of the Delphi study, involving 24 of the invited 32 expert panelists, a strong consensus was achieved across all components of the ERAS protocol tailored to resource‐limited settings (see Tables 4 and 5, Supporting Information S2: Appendix 2 for survey). Nearly, all proposed components from round one received 100% agreement. Consensus of novel interventions proposed and included are summarized in (Figure 1).

**TABLE 4:** Round two results assessing specific recommendations for a preoperative pathway of elective GI and HPB surgery patients at tertiary hospitals in Ethiopia.

Component		Include unmodified	Include but modify	Include elsewhere in the protocol	Exclude	Agreement (%)	Consolidated suggestions
Preoperative	Preadmission information, education, and counseling	18	6	—	—	100	Surgeons should clearly explain the procedure, risks, and alternatives. In the absence of dedicated ERAS nurses, a defined backup education plan should be in place.
	Optimization (smoking, alcohol, HIV, and anemia)	17	7	—	—	100	Routine HIV screening is acceptable; avoid delays from CD4/viral load determination unless essential. Correct viral load threshold to < 10,000 copies. Clarify target Hb, iron therapy type, and duration (≥ 4 weeks—noncancer, ≥ 1 week—cancer). Adjust alcohol abstinence advice for withdrawal risk. Include urgent/emergent surgery exceptions.
	Preoperative nutritional care	19	5	—	—	100	Assess nutrition using the ABCD approach (anthropometric, biochemical, clinical, and dietary). Use locally available alternatives to commercial nutrition supplements.
	Selective mechanical bowel prep (MBP)	10	14	—	—	100	In high‐fiber diet settings, MBP + oral antibiotics may reduce technical difficulties. Consider surgeon preference and local diet. Favor selective over routine use.
	Preoperative fasting and CHO loading	19	5	—	—	100	Maintain 6h solids/2h clear fluids fasting. Carbohydrate loading and optional robust evidence lacking. Delay oral intake in patients with gastric stasis.
	Preoperative fluid and electrolyte therapy	22	2	—	—	100	Replace NS with balanced fluids. Recommend specific fluid types and guide volumes.
	PONV prophylaxis	22	2			100	List the types of antiemetics along with their dosages, and categorize them into first‐line and second‐line treatment options.
	Functional status/frailty assessment	17	5	1	1	91.6	Separate frailty from functional status; apply tools such as ECOG universally. In low‐resource settings, prioritize basics (nutrition, physiotherapy, and anemia) first. Use ASA to stratify risk.
Intraoperative	Surgical safety checklist	22	2	—	—	100	Specify use of the WHO checklist.
	Antimicrobial prophylaxis	24	—	—	—	100	—
	VTE prophylaxis	22	2	—	—	100	Extend prophylaxis to 28 days postop in cancer cases. Address implementation barriers (drug/stocking access).
	Standard anesthesia protocol‐ short‐acting anesthetic agents, lung‐protective ventilation, and complete reversal of neuromuscular blockade	23	1	—	—	100	—
	Normothermia (active/passive)	23	1	—	—	100	—
	Multimodal opioid‐sparing analgesia	20	4	—	—	100	Limit TEA to centers with trained personnel. Paraspinal blocks are viable, low‐cost alternatives. Availability and monitoring capacity should guide choice.
	Fluid balance: Goal‐directed fluid therapy (GDFT)	22	2	—	—	100	Continue IV fluids postop based on ileus risk. Low BP/HR limits may help.
Postoperative	Postop fluid and electrolyte therapy	19	5	—	—	100	Recommend maintenance fluids with or without glucose (e.g., 1/2 strength normal saline) and adjust based on individual needs to achieve a sodium goal of approximately 1 mmol/kg/day.
	Postop nutritional care	17	7			100	Enteral feeding if no nausea/vomiting. Avoid rigid timelines—tailor to intraop findings. Feeding jejunostomy should be routine in esophagectomy. For upper GI/cancer surgeries, delay oral intake if needed.
	Early structured mobilization	23	1	—	—	100	Define ambulation targets (e.g., sit/walk for several hours/day). Consider neuromodulators, such as gabapentin, to support pain and mobilization.
	Postop analgesia/opioid minimization	20	4	—	—	100	Avoid TEA in liver surgery Avoid NSAIDs in patients at risk for anastomotic leak.
	Postop ileus prevention	22	1	1	—	100	Consider chewing gum as a GI stimulant.
	Discharge criteria	23	1	—	—	100	Discharge readiness should include adequate oral intake (fluids, calories, and protein) not just a “normal diet”.

Abbreviations: ASA: American Society of Anesthesiologists, BP: blood pressure, ECOG: Easter Cooperative Oncology Group, GI: gastrointestinal, Hb: hemoglobin, HIV: human immunodeficiency virus, HR: heart rate, MBP: mechanical bowel preparation, NS: normal saline, NSAIDs: nonsteroidal anti‐inflammatory drugs, TEA: thoracic epidural analgesia, WHO: World Health Organization.

**TABLE 5:** Comprehensive ERAS protocol with delphi consensus, evidence, and GRADE of recommendation.

Phase	Component	Evidence level	GRADE recommendation
Preoperative	Preadmission information, education, and counseling	Moderate –low high	Strong
	Assessment of functional status or frailty	Low	Strong
	Optimization (smoking/alcohol cessation, HIV, and anemia)	Moderate–high	Strong
	Preoperative nutritional care	Moderate–low	Strong
	Selective mechanical bowel preparation	Moderate	Strong
	Fasting and carbohydrate loading	Moderate–high	Strong
	Fluid and electrolyte therapy	Moderate	Strong
	PONV prophylaxis	High	Strong
	Preoperative respiratory exercise	Low–moderate	Strong
	Preoperative optimization of OJ		
	Establishment of hospital ERAS team		
	Continuous medical education (CME) on ERAS		
Intraoperative	Surgical safety checklist	High	Strong
	Antimicrobial prophylaxis	High	Strong
	VTE prophylaxis	High	Strong
	Standard anesthesia protocol	Moderate	Strong
	Maintenance of normothermia	Moderate–high	Strong
	Opioid‐sparing multimodal analgesia	High	Strong
	Intraoperative fluid therapy	High	Strong
	Transfusion guidelines and strategies to minimize blood loss		
Postoperative	Postoperative fluid and electrolyte therapy	High	Strong
	Postoperative nutritional care (early feeding)	High	Strong
	Early mobilization	Moderate	Strong
	Multimodal analgesia	High	Strong
	Opioid minimization	High	Strong
	Prevention of ileus (TEA, no NG tube, and limited IV fluids)	Moderate–high	Strong
	Discharge criteria	Moderate–high	Strong
	Protocol audit and feedback mechanism	High	Strong

Abbreviations: CME: continuing medical education, ERAS: enhanced recovery after surgery, HIV: human immunodeficiency virus, IV: intravenous, NG: nasogastric, OJ: obstructive jaundice, PONV: postoperative nausea and vomiting, TEA: thoracic epidural analgesia, VTE: venous thromboembolism.

**FIGURE 2:**
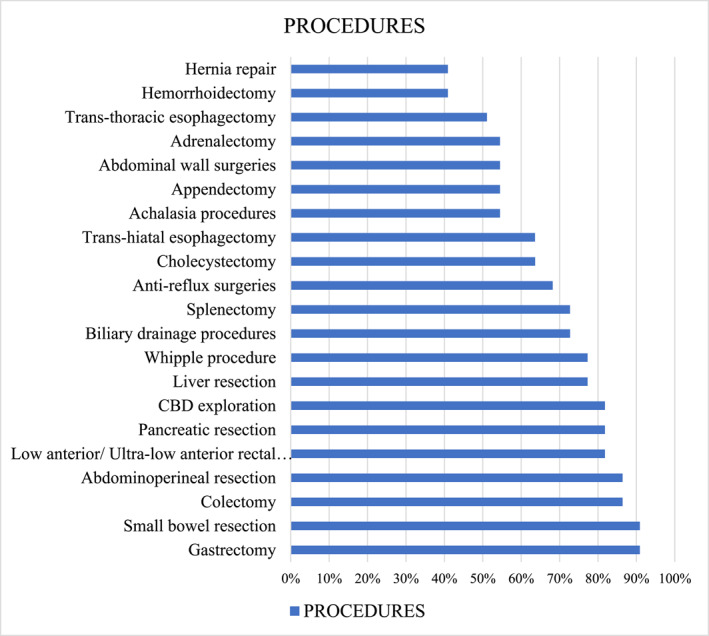
Procedures for which the protocol can be applied.

In the second phase of the Delphi process, expert panelists were also asked which procedures should be included in the proposed ERAS adaptation (see Figure 2 and Supporting Information S3: Appendix 3). Panelists reached a strong consensus on the inclusion of seven major abdominal and thoracic surgical procedures for standardized protocol application, with agreement levels equal to or exceeding 80%.

## 4 Discussion

This Delphi study successfully established expert consensus on ERAS components tailored to GI and HPB surgical care in Ethiopian tertiary hospitals. Strong expert consensus demonstrates a unified commitment among local surgical, and anesthesia experts to adapt evidence‐informed perioperative care pathways contextualized to LIC settings. The results align with key principles established by the ERAS Society, while adapting flexibly to the region's resource realities and disease burden. The protocol contains a total of 27 elements: 8 preoperative, 7 intraoperative, 6 postoperative, and 6 novel elements suggested by the panelists (Table 5). The findings strongly align with core ERAS Society guidelines for LMICs.

### 4.1 Preoperative

The expert panel highlighted the critical importance of comprehensive preoperative patient education, particularly in low‐resource settings where dedicated ERAS coordinators or specialized nursing staff are often unavailable. Structured patient education is shown to reduce anxiety, improve compliance, and enhance recovery outcomes [2, 3]. In such contexts, surgeon‐led counseling was emphasized as a practical and impactful intervention. The panel advised separating the assessments for preoperative frailty and functional status and applying practical context‐appropriate tools such as the Eastern Cooperative Oncology Group (ECOG) performance status and the American Society of Anesthesiologists (ASA) physical status classification [12, 13]. Basic frailty assessments are predictive of postoperative outcomes and are feasible in resource‐limited environments [14, 15, 16, 17, 18, 19].

For nutritional evaluation and optimization, the panel recommended the ABCD approach (anthropometric, biochemical, clinical, and dietary), with an emphasis on using affordable locally available nutritional substitutes in place of expensive commercial supplements. This aligns with ESPEN guidelines, which advocate for early identification and correction of malnutrition before surgery [20]. Unlike Western ERAS guidelines that discourage routine mechanical bowel preparation (MBP) due to risks of dehydration and patient discomfort, the panel supported its contextual inclusion in colorectal surgery, particularly for left‐sided resections and cases involving stenosing lesions [21, 22, 23]. This recommendation is based on the high‐fiber content of local diets, late‐stage tumor presentations, and evidence from recent literature supporting the use of MBP combined with oral antibiotics in reducing surgical site infections [2, 3, 24]. The panel advocated for a modified context‐sensitive approach rather than outright exclusion of MBP.

Standardization of preoperative fasting was recommended. Although carbohydrate loading is supported in global ERAS protocols [2, 3, 23], the panel suggested it remain optional due to variable feasibility and inconclusive local evidence.

### 4.2 Intraoperative

There was a unanimous consensus among the Delphi panelists on the mandatory use of the WHO Surgical Safety Checklist, reaffirming its critical role in improving perioperative communication, enhancing team coordination, and reducing avoidable surgical errors and complications [25, 26, 27, 28].

For VTE prophylaxis, the panel recommended extending prophylaxis to 28 days postoperatively in patients undergoing cancer surgery. This aligns with global surgical oncology guidelines, which support extended‐duration prophylaxis due to the persistently elevated thrombotic risk in this population [25, 26, 29, 30]. However, panelists acknowledged practical limitations in access to low‐molecular‐weight heparin (LMWH) or newer anticoagulants in low‐resource settings and emphasized the importance of individualized risk‐benefit decisions where cost and availability pose barriers.

The panel has strongly agreed on the intraoperative and postoperative maintenance of normal body temperature > 36°C. Warming blankets, warm intravenous (IV) fluids, blood components, and radiant warmers can be used for maintaining intraoperative normothermia in the Ethiopian setting [27, 28, 31].

Although epidural analgesia remains an effective option for major open abdominal surgeries, its recommendation was limited to centers with trained anesthesiologists and appropriate monitoring capacity, acknowledging the technical demands and risk of complications in low‐resource settings. It may not always be appropriate for major liver surgery. Paraspinal or transverse abdominis plane (TAP) blocks were recommended, given their safety, simplicity, low cost, and findings supported by recent trials comparing them favorably with more invasive regional techniques [32, 33, 34, 35, 36].

### 4.3 Postoperative

Postoperatively, ongoing tailored intravenous fluid administration was advised, supported by evidence linking restrictive individualized fluid strategies with reduced postoperative complications and faster gastrointestinal recovery [9, 37, 38, 39, 40]. The panel recommended that postoperative IV fluids be discontinued based on clearly defined clinical goals, typically between postoperative days two and three. Patients should be encouraged to drink when fully recovered and offered an oral diet within 4 h after abdominal surgery. This individualized approach aligns with fluid stewardship principles in ERAS protocols, which discourage indiscriminate IV fluid administration and emphasize early return to oral intake where possible [2, 3, 37, 39, 40].

The importance of early enteral nutrition was reaffirmed, with panelists recommending flexible initiation as soon as nausea or vomiting resolves rather than rigid adherence to fixed timelines. This position reflects both ERAS Society recommendations and meta‐analyses showing that early feeding reduces infectious complications and shortens hospital stays without increasing the risk of anastomotic leakage [2, 3].

Regarding mobilization, the panel emphasized the need for structured and measurable goals, such as sitting up or walking for several hours each day, to promote functional recovery and reduce complications such as pneumonia and thromboembolism [2, 41, 42]. Pain control was seen as central to this effort, with neuromodulatory agents, such as gabapentin, recommended as adjuncts to reduce opioid requirements. This is supported by literature demonstrating that multimodal analgesia, including gabapentinoids, improves pain control and facilitates early mobilization [42].

For postoperative analgesia, the combination of paracetamol and nonsteroidal anti‐inflammatories is recommended as baseline multimodal analgesics unless a specific contraindication exists. It has been also suggested that long‐acting regional nerve blocks were proposed as safer and effective alternatives to TEA where available [2, 3, 36, 42].

To address postoperative ileus, the panel endorsed the use of chewing gum as a low‐cost evidence‐based intervention that stimulates gastrointestinal motility. This practice has been shown in multiple RCTs to reduce time to bowel movement after abdominal surgery [43]. Regarding discharge readiness, the panel recommended moving beyond the simplistic criterion of tolerating a “normal diet” and instead proposed objective nutritional benchmarks, including sufficient intake of fluids, calories, and proteins.

### 4.4 Novel Elements Included in the ERAS Protocol

Among the eight novel interventions introduced in the first round of the Delphi consensus, six achieved strong expert agreement for inclusion. The highest level of agreement was for the implementation of regular audit mechanisms. Audit and feedback are critical to sustaining ERAS compliance and improving patient outcomes [44, 45]. A multicenter implementation study demonstrated that consistent ERAS auditing led to a 50% reduction in length of stay and significant adherence improvements across perioperative domains. Additionally, audit‐driven feedback loops are strongly endorsed in the ERAS Implementation Program (EIP) model, which emphasizes iterative quality improvement [2, 3].

The recommendation to establish an ERAS team echoes successful implementation strategies in both high‐ and low‐resource environments. For example, the ERAS Interactive Audit System (EIAS) initiative demonstrated that institutions with formal multidisciplinary ERAS teams achieve higher compliance rates and better outcomes [44, 45, 46]. Multidisciplinary ERAS teams help overcome task fragmentation and role ambiguity, challenges commonly seen in resource‐limited settings [47].

The panel endorsed preoperative respiratory exercises reflecting accumulating evidence supporting their role in reducing pulmonary complications [48]. In LMICs, limited resources increase postoperative respiratory risks, making simple interventions like breathing exercises highly valuable.

In patients with obstructive jaundice, correcting fluid and electrolyte imbalances, replenishing vitamin K to reduce bleeding risk, and giving appropriate antibiotics are key to preventing renal complications, prolonged ICU stay, and delayed recovery. The panel emphasized that this is particularly important in LMICs, where patients often present late with advanced disease [49].

The panel also agreed that, beyond correcting anemia, adopting transfusion guidelines and blood conservation strategies, such as restrictive thresholds (Hb 7–8 g/dL) and antifibrinolytics, supports ERAS principles and helps reduce complications, ICU stay, and recovery time [50, 51].

Finally, the integration of continuous medical education (CME) on ERAS principles was supported [2, 52]. In low‐resource settings, where perioperative training is often limited, CME programs can play a vital role in disseminating ERAS knowledge and updating practices.

### 4.5 Procedures for Protocol Application

Experts reached strong consensus (≥ 80%) to include seven major abdominal procedures in this ERAS protocol (Figure 1). Selection was based on complexity, educational value, and relevance in the management of both benign and malignant GI and HPB conditions. The consensus suggests that these procedures are considered essential for developing core surgical competencies. Conversely, procedures falling below the 80% threshold were not deemed mandatory for inclusion, likely reflecting variability in institutional exposure, case volume, or subspecialty focus.

### 4.6 Limitations and Strengths

Although the Delphi method provides expert consensus, it does not substitute for prospective clinical validation. It is important to note that this protocol adaptation was carried out as part of an implementation strategy within an ongoing project at Jimma University Medical Center, a tertiary teaching hospital in Ethiopia. We plan to revise the protocol based on insights gained from the implementation process to better align with the local context. Despite this limitation, the study's strength lies in its systematic approach to consensus‐building, incorporation of multidisciplinary perspectives, and strong agreement among participants demonstrating the potential for ERAS protocol standardization even in resource‐constrained environments.

Another limitation of this study is the expert panel's composition as some members had limited expertise in certain areas, which may have influenced item selection. Nurses were not included due to limited availability of perioperative specialists and low ERAS awareness in our setting; however, input from a nursing–pharmacy professional specializing in pain management was obtained to contextualize protocol components.

Another limitation of the study is the attrition of panelists during the second round of the Delphi process. Although the drop‐in participation was not substantial, it may still have inflated the apparent level of consensus and introduced some bias into the guideline. This reduction in participation could also have affected the representativeness of the expert panel, especially given that the original group was purposively selected to ensure a diverse mix of skills, experiences, and perspectives relevant to the topic.

Finally, these key elements pertain to various subspecialties and may not capture the specific nuances of every clinical condition. The content of this document is not intended to replace clinical judgment and should be applied alongside appropriate clinical guidelines.

## 5 Conclusion

This study represents a critical step toward implementing standardized resource‐appropriate ERAS pathways for GI and HPB surgeries in Ethiopia. By aligning with international best practices and integrating pragmatic adaptations, the protocol offers a model for scalable ERAS implementation in LMICs. With structured rollout, ongoing audit, and periodic updates, it has the potential to improve surgical outcomes, reduce variation in care, and establish a sustainable culture of perioperative excellence in the region. After the implementation of this protocol, continuous updates and improvements will be made based on emerging evidence and best practices.

## Author Contributions

**Wongel Tena Shale:** conceptualization, data curation, formal analysis, investigation, methodology, project administration, resources, supervision, validation, visualization, writing – original draft, writing – review and editing. **Abraham Teshome Sahilemariam:** conceptualization, data curation, formal analysis, investigation, methodology, validation, visualization, writing – original draft, writing – review and editing. **Tilahun Habte Nureta:** conceptualization, data curation, investigation, methodology, validation, writing – review and editing. **Tadesse Girma Moges:** conceptualization, data curation, investigation, methodology, validation, writing – review and editing. **Edosa Kejela Keno:** conceptualization, investigation, methodology, validation, writing – review and editing. **Wondu Reta Demissie:** conceptualization, investigation, methodology, validation, writing – review and editing. **Robert K. Parker:** conceptualization, investigation, methodology, validation, writing – review and editing. **Mercedes Pilkington:** conceptualization, investigation, methodology, supervision, validation, visualization, writing – original draft, writing – review and editing.

## Funding

The current work had no funding. However, it is part of an implementation project which is funded by Fenot associates, COSECSA‐RCSI global surgery research collaboration project, CHESA–UCSF, and Jimma University.

## Ethics Statement

Ethical approval was obtained from Jimma University Institutional review board to conduct the study. Informed consent was obtained from all individual participants included in the study. Data were deidentified and anonymized before the presentation. Panelists were given the option to be identified as contributors.

## Conflicts of Interest

The authors declare no conflicts of interest.

## Supporting information


Supporting Information S1

Supporting Information S2

Supporting Information S3

## Data Availability

The data that support the findings of this study are available from the corresponding author upon request. The data are not publicly available due to privacy or ethical restrictions.
