# Supplementary material for: Adaptation of Enhanced Recovery After Surgery Protocol for Elective Gastrointestinal and Hepatopancreaticobiliary Surgeries for Tertiary Hospitals in Ethiopia: A Modified Delphi Study
Source: World J Surg. 2026 Mar 2;50(4):860–73. doi: 10.1002/wjs.70274 (PMC13070447; doi:10.1002/wjs.70274)
Supplement: Supplementary file 1 — Supporting Information S1 [file WJS-50-860-s002.docx]

**Questionnaire for Delphi consensus**

**Round one survey**

**Letter of Information and Implied Consent Form**

**Principal Investigator:** Dr.Wongel Tena Shale, Assistant Professor, Department of Surgery, Jimma University, Ethiopia, [tenawongel@gmail.com](mailto:tenawongel@gmail.com), [wongel.tena@ju.edu.et](mailto:wongel.tena@ju.edu.et)

**Co-Investigators**: Dr. Abraham Teshome Sahilemariam, Dr. Tilahun Habte Nureta, Dr. Tadesse Girma Moges

**Purpose:** You are asked to participate in the development of an enhanced recovery after surgery (ERAS) protocol utilizing a modified Delphi consensus method for adult patients undergoing elective gastrointestinal and hepatobiliary surgeries at tertiary hospitals in Ethiopia.

**Study Procedures:** If you are willing to take part, kindly finish this initial survey as it is a modified Delphi study. It should only take you ten minutes or less to finish this. This survey is the first of two or more rounds that will be used to establish the consensus among experts. You will be asked to share your thoughts on the best ways to adapt the current ERAS guidelines for the context we have in tertiary hospital in Ethiopia. You are not required to respond to any questions. After submitting this survey, you will receive an invitation to take part in future rounds. If you haven't finished the pertinent survey, you will receive a reminder one week after the invitation.

**Project Outcomes:** The goal of the project is to develop a consensus ERAS protocol that may be used in low- and middle-income countries (LMICs) for adult patients having elective GI and HPB surgeries to treat GI and HPB surgical conditions. The experts involved will decide on the final content and level of unanimity. The study's conclusions could be published in an academic journal.
**Potential Benefits:** Participation may not directly benefit you in any way. In the future, patients could ideally benefit from the development of enhanced recuperation guidelines that are contextually suitable. You will be given the option to be identified as a contributor when the final protocol gets published, and if you choose so you will be acknowledged as a contributor.

**Potential Risks:** None anticipated. You may withdraw your participation at any time. If study results are published, all data will be de-identified and consensus presented in aggregate. Publication may require open access to the de-identified data in which case you will not be able to withdraw your data after publication. All study data will be password protected and be stored on a private, encrypted, password-protected computer.

**Confidentiality:** The final consensus document will indicate the degree of agreement, but it won't include your name or your reaction to any particular element. This initial survey will gather your name, professional role, and email address; these details will be kept on file to make it easier for you to be invited to participate in subsequent rounds of surveys. The Principal Investigator and the study team will be the only ones with access to the research data and your contact details. However, you will be given the option to be identified as a contributor when the final protocol gets published.

**Remuneration/Compensation:** We will not pay you for participating.
**Contact for information:** If you have any questions or concerns, please contact
Dr. Wongel or the study PI. The names and email addresses are listed at the top of the first page of this form.

**Consent**: There is no obligation for you to participate in this study; you are free to decline or leave at any moment. The Modified Delphi Consensus first round will commence after you click the forward arrow below, indicating your willingness to participate as described above. We'll assume that you still consent to continue giving us your opinions through the survey. Any member of the study team or the principal investigator can be contacted at any time to request a withdrawal.

In order to improve surgical outcomes, enhanced recovery after surgery (ERAS) protocols usually include 10–30 evidence-based suggestions that can be put into practice during the perioperative phase. Sample guidelines can be found at **ERASSociety.org.**
You are being asked to contribute to the development of an enhanced post-operative recovery guideline for adults undergoing elective gastrointestinal and hepatobiliary surgery at tertiary centres in Ethiopia.

You will be asked to rank the significance of each possible element's inclusion or exclusion in an ERAS guideline that would be applied particularly in low-resource environments.

We kindly invite those of you who operate in environments with fewer resources to think about whether each element could or should be used there. For those in a high resource setting, consider which you think would be important and feasible to include/exclude in a LIC. Please rate each item on a 5-point Likert scale with 1 = must exclude and 5 = must include. For each proposed component, you will be invited to provide any recommendations around modifications to improve the feasibility, efficacy, or acceptability in an LMIC (optional). Finally, you will be asked to submit any additional ideas (even new recommendations that had never been included in anyone of the existing ERAS guidelines so far) for inclusion that might be pertinent in view of our context.

1. **Which of these PREOPERATIVE components do you think should be included in
   an ERAS Protocol adapted for elective GI and HPB surgery patients at tertiary centres in Ethiopia**

*Note: You are not required to consider what the recommendation itself will be, just
whether you think it would be important to consider a recommendation about this
topic.*

|  | 1-Must Exclude | 2-Should Exclude | 3-neutral | 4-Should Include | 5-Must Include |
| --- | --- | --- | --- | --- | --- |
| 1. Preadmission information, education and counselling |  |  |  |  |  |
| 1. Optimization/Prehabilitation: Smoking cessation, Alcohol, routine HIV screening (If +ve, CD4, Viral load), anemia (Iron supplementation), HTN, DM, Malnutrition, Delirium |  |  |  |  |  |
| 1. Preoperative nutritional care |  |  |  |  |  |
| 1. Selective mechanical bowel preparation |  |  |  |  |  |
| 1. Preoperative fasting and carbohydrate loading |  |  |  |  |  |
| 1. Preoperative fluid and electrolyte therapy |  |  |  |  |  |
| 1. Preoperative biliary drainage in cholestatic liver |  |  |  |  |  |
| 1. Premedication: selective anxiolytics |  |  |  |  |  |
| 1. PONV prophylaxis |  |  |  |  |  |
| 1. Preoperative administration of steroids for planned liver resection |  |  |  |  |  |
| 1. Hair removal: clipper |  |  |  |  |  |

1. **Please provide any suggestions for modification or additions for the preoperative setting:**

**-----------------------------------------------------------------------------------------------------------------------------------------------------------------------------------------------------------------------------------------------------------------------------------------------------------------------------------------------------------------------------------------------------------------------------------------------------------------------------------------------------------------------------------**

1. **Which of these INTRAOPERATIVE components do you think should be included in an ERAS Protocol adapted for elective GI and HPB surgery patients at tertiary centres in Ethiopia?**

*Note: You are not required to consider what the recommendation itself will be, just
whether you think it would be important to consider a recommendation about this
topic.*

|  | 1-Must Exclude | 2-Should Exclude | 3-neutral | 4-Should Include | 5-Must Include |
| --- | --- | --- | --- | --- | --- |
| 1. Surgical safety checklist |  |  |  |  |  |
| 1. Antimicrobial prophylaxis |  |  |  |  |  |
| 1. Venous thromboembolism (VTE) prophylaxis: compression stocking and/or intermittent pneumatic compression together with either a LMWH or unfractionated heparin |  |  |  |  |  |
| 1. Standard anaesthesia protocol: Short-acting anaesthetic agents, lung-protective ventilation, and complete reversal of neuromuscular blockade |  |  |  |  |  |
| 1. Normothermia: active Vs Passive warming |  |  |  |  |  |
| 1. Multimodal opioid sparing analgesia: Short-acting opioid sparing analgesia combined with local and regional blocks. TEA, TAP/RA/ Subcostal blocks, epidural catheter, wound catheter |  |  |  |  |  |
| 1. Fluid balance: Near- zero fluid balance. Goal-directed fluid therapy (GDFT). |  |  |  |  |  |
| 1. Surgical access: Open Vs MIS   Different types of incision for open surgery |  |  |  |  |  |
| 1. Nasogastric tubes (NGT) |  |  |  |  |  |
| 1. Drains (peritoneal cavity and pelvis) |  |  |  |  |  |

1. **Please provide any suggestions for modification or additions for the
   intraoperative setting:**

**------------------------------------------------------------------------------------------------------------------------------------------------------------------------------------------------------------------------------------------------------------------------------------------------------------------------------------------------------------------------------------------------------------------------------------------------------------------------------------------------------------------------------------------------------------**

1. **Which of these POSTOPERATIVE components do you think should be included in an ERAS Protocol adapted for elective GI and HPB surgery patients at tertiary centres in Ethiopia?**

*Note: You are not required to consider what the recommendation itself will be, just
whether you think it would be important to consider a recommendation about this
topic.*

|  | 1-Must Exclude | 2-Should Exclude | 3-neutral | 4-Should Include | 5-Must Include |
| --- | --- | --- | --- | --- | --- |
| 1. Postoperative fluid and electrolyte therapy |  |  |  |  |  |
| 1. Postoperative nutritional care |  |  |  |  |  |
| 1. Early structured mobilisation plan |  |  |  |  |  |
| 1. Postoperative analgesia |  |  |  |  |  |
| 1. Urinary drainage |  |  |  |  |  |
| 1. Prevention of postoperative ileus |  |  |  |  |  |
| 1. Postoperative glycaemic control and intensive insulin treatment in the ICU |  |  |  |  |  |

1. **Please provide any suggestions for modification or additions for the
   postoperative setting:**

------------------------------------------------------------------------------------------------------------------------------------------------------------------------------------------------------------------------------------------------------------------------------------------------------------------------------------------------------------------------------------------------------------------------------------------------------------------------------------------------------------------------------------------------------------------------------------------------------------------------------------------------

1. **Please provide any additional areas you think should be considered in creating
   an ERAS protocol adapted for elective GI and HPB surgery patients at tertiary centres in Ethiopia**

**------------------------------------------------------------------------------------------------------------------------------------------------------------------------------------------------------------------------------------------------------------------------------------------------------------------------------------------------------------------------------------------------------------------------------------------------------------------------------------------------------------------------------------------------------------------------------------------------------------------------------------------------------------------------**

1. **Please provide your contact information so that you can be included in the list
   of contributors and for the second round of the Modified Delphi consensus**
2. **Name: _____________________________________________**
3. **Email address: ______________________________________**
4. **Profession: ___________________________________________**
5. **Years of experience: ____________________________________**
6. **Country: ____________________________________________**
