# Supplementary material for: Adaptation of Enhanced Recovery After Surgery Protocol for Elective Gastrointestinal and Hepatopancreaticobiliary Surgeries for Tertiary Hospitals in Ethiopia: A Modified Delphi Study
Source: World J Surg. 2026 Mar 2;50(4):860–73. doi: 10.1002/wjs.70274 (PMC13070447; doi:10.1002/wjs.70274)
Supplement: Supplementary file 2 — Supporting Information S2 [file WJS-50-860-s003.docx]

You are asked to participate in the development of an enhanced recovery after surgery (ERAS) protocol, utilizing a modified Delphi consensus method for adult patients undergoing elective gastrointestinal and hepatopancreatic-biliary surgeries at tertiary hospitals in Ethiopia.

**Round two survey**

**Letter of Information and Implied Consent Form**

**Principal Investigator:** Dr. Wongel Tena Shale, Assistant Professor, Department of Surgery, Jimma University, Jimma, Ethiopia, [tenawongel@gmail.com](mailto:tenawongel@gmail.com), [wongel.tena@ju.edu.et](mailto:wongel.tena@ju.edu.et)

**Co-Investigators**: Dr. Abraham Teshome Sahilemariam, Dr. Tilahun Habte Nureta, Dr. Tadesse Girma Moges, Dr. Robert K Parker, Dr. Mercedes Pilkington

**Purpose:** You are asked to participate in the development of an enhanced recovery after surgery (ERAS) protocol utilizing a modified Delphi consensus method for adult patients undergoing elective gastrointestinal (GI) and hepatopancreatic-biliary (HPB) surgeries at tertiary hospitals in Ethiopia.

**Study Procedures:** If you are willing to take part, kindly finish this second round of questions as it is a modified Delphi study. It should only take you 30 minutes or less to finish this. This survey is the second of two or more rounds that will be used to establish a consensus among experts. You will be asked to share your thoughts on the best ways to adapt the current ERAS guidelines for the context we have in tertiary hospitals in Ethiopia. After submitting this survey, you will be notified if future rounds are warranted. If you haven't finished the pertinent survey, you will receive a reminder one week after the invitation.

**Project Outcomes:** The goal of the project is to develop a consensus ERAS protocol that may be used in low-income countries like Ethiopia for adult patients having elective GI and HPB surgeries to treat GI and HPB surgical conditions. The experts involved will decide on the final content and level of unanimity. The study's conclusions could be published in an academic journal.

**Potential Benefits:** Participation may not directly benefit you in any way. In the future, patients could ideally benefit from the development of enhanced recovery guidelines that are contextually suitable. You will be given the option to be identified as a contributor when the final protocol gets published, and if you choose so you will be acknowledged as a contributor.

**Potential Risks:** None anticipated. You may withdraw your participation at any time. If study results are published, all data will be de-identified, and consensus presented in aggregate. Publication may require open access to the de-identified data in which case you will not be able to withdraw your data after publication. All study data will be password protected and be stored on a private, encrypted, password-protected computer.

**Confidentiality:** The final consensus document will indicate the degree of agreement, but it will not include your name or your reaction to any particular element. The Principal Investigator and the study team will be the only ones with access to the research data and your contact details. However, you will be given the option to be identified as a contributor when the final protocol gets published.

**Remuneration/Compensation:** We will not pay you for participating.

**Contact for information:** If you have any questions or concerns, please contact Dr. Wongel or the study PI. The names and email addresses are listed at the top of the first page of this form.

**Consent**: There is no obligation for you to participate in this study; you are free to decline or leave at any moment. The Modified Delphi Consensus second round will commence after you click the forward arrow below, indicating your willingness to participate as described above. We'll assume that you still consent to continue giving us your opinions through the survey. Any member of the study team or the principal investigator can be contacted at any time to request a withdrawal.

To improve surgical outcomes, enhanced recovery after surgery (ERAS) protocols usually include 10–30 evidence-based suggestions that can be put into practice during the perioperative phase. Sample guidelines can be found at **ERASSociety.org.**

You are being asked to contribute to the development of an enhanced peri-operative recovery guideline for adults undergoing elective gastrointestinal and hepatopancreatic-biliary surgery at tertiary centers in Ethiopia.

**You will be required to read the specific recommendations made for the components that were accepted for inclusion in accordance with the first round's consensus. *Include the recommendation as given, include but modify, include elsewhere in the protocol, and exclude*  are your four options. You will be prompted to provide an explanation if you decide to include but modify or exclude. Additionally, you will be asked to rank the importance of including or excluding additional Nobel elements (found in the round one responses) from an ERAS guideline that might be used, especially in low-resource situations.** Please rate each item in section 3 of the questioner, on a 5-point Likert scale with 1 = must exclude and 5 = must include. Finally, you will be asked to submit any additional ideas that might be pertinent in view of our context.

**Section 1: Procedures for which the protocol can be applied**

Q1: Which of these Procedures do you recommend to be included for the adapted ERAS Protocol application in elective GI and HPB surgery patients at tertiary hospitals in Ethiopia?

1. Trans-thoracic esophagectomy

1. Trans-hiatal esophagectomy
2. Achalasia procedures
3. Anti-reflux surgeries
4. Gastrectomy
5. Small bowel resection
6. Appendectomy
7. Right colectomy
8. Left colectomy
9. Low anterior/ Ultra-low anterior rectal resection
10. Abdominoperineal resection
11. Hemorrhoidectomy
12. Hernia repair
13. Abdominal wall surgeries
14. Splenectomy
15. Adrenalectomy
16. Liver resection
17. Pancreatic resection
18. Whipple procedure
19. Cholecystectomy
20. CBD exploration
21. Biliary drainage procedures

Q2: Do you have any additional comments regarding the type of procedures for applying the adapted ERAS protocol?

…

**Section 2: Specific recommendations**

In the first round, your expert opinions were taken into account to decide the scope of the adapted ERAS protocol we are creating for the Ethiopian tertiary hospital setups in hepatobiliary surgeries (HPB), and gastrointestinal (GI) surgeries. Based on the 80% consensus priori we set, 22 out of 30 elements made the cut overall. i.e. 8, 7, and 7 elements in the preoperative, intraoperative, and postoperative components respectively. In the second round, specific recommendations are proposed based on the best available evidence in the literature. We have placed summary boxes attached to each element explaining the available evidence briefly. We have also put together some relevant citations for your reference. Consensus for inclusion will be sought with a 4-point Likert scale (1-Include unmodified, 2- Include but further modify content, 3- Include elsewhere in the protocol, and 4- Exclude from protocol). If you choose to include but further modify or exclude from protocol, you will be asked to further elaborate your response.

2.1. Specific Recommendations for Preoperative Components

2.1.1. Preadmission information, education, and counseling

Evidence box

*Preadmission education and counseling are crucial for patients and their caregivers, aiming to reduce anxiety related to anesthesia and surgery while improving preparedness and satisfaction. This education should be provided in various formats—oral, written, and pictorial—by an ERAS nurse coordinator soon after the decision to operate. In low- and middle-income countries (LMICs), challenges such as limited access to healthcare professionals may require elected healthcare workers to fulfill this role.*

Recommendation: Pre-admission patient education and counseling should be given to all patients on the day of decision for elective surgery at the admission clinic. It is preferably given by an ERAS coordinator nurse to the patient and a family member/caregiver. The education should be provided in various formats—oral, written, and pictorial. The content should cover

- detailed, procedure-specific, and patient-centered information
- Offer psychological support to alleviate fears and enhance recovery
- What to expect post-surgery, pain management, post-operative phase (deep breathing exercise, wound care), and addressing any body image disturbance.
- Discharge plan

What is your response to the above recommendation on Pre-admission patient education?

1. Include unmodified
2. Include but modify
3. Include elsewhere in the protocol
4. Exclude

Citation

1. Gustafsson UO, Scott MJ, Hubner M, Nygren J, Demartines N, Francis N, Rockall TA, Young-Fadok TM, Hill AG, Soop M, De Boer HD. Guidelines for perioperative care in elective colorectal surgery: Enhanced Recovery After Surgery (ERAS®) Society recommendations: 2018. World journal of surgery. 2019 Mar 15;43:659-95.
2. Oodit, Ravi, et al. "Guidelines for perioperative care in elective abdominal and pelvic surgery at primary and secondary hospitals in low–middle-income countries (LMICs): enhanced recovery after surgery (ERAS) society recommendation." *World Journal of Surgery* 46.8 (2022): 1826-1843.
   - 1. Assessment of functional status or frailty

Evidence box

*The increasing number of elderly individuals requiring surgery is a result of advancements in medical treatments and longer life expectancies. However, reduced physical function and frailty are significant predictors of poor surgical outcomes in this population. The 30-day mortality rate among the oldest patients remains high, contributing to an elevated 1-year mortality rate. Nearly 40% of elderly patients may face postoperative complications, with serious complications occurring in 45% of those cases. Surgery poses particular risks for frail patients, potentially leading to irreversible functional decline, especially given their higher prevalence of comorbidities. Despite these challenges, research indicates that older patients can benefit from Enhanced Recovery After Surgery (ERAS) protocols, achieving outcomes similar to younger patients when treatment plans are tailored to their biological characteristics rather than just their chronological age.*

Recommendation: Frailty or functional status should be considered as an essential factor to evaluate in preoperative assessment.

- Scoring: There is no gold standard for detecting frailty. Based on available resources and expertise any of the scoring systems can be used. Some of the most widely used, technically simpler scoring mechanisms include; the **Fried Frailty Phenotype, Clinical Frailty Scale (CFS), Edmonton Frail Scale (EFS)**
- ERAS protocol is recommended to be applied in elderly and frail patients with special emphasis on the following areas.
  - A multidisciplinary team approach involving a geriatrician is recommended if expertise is available
  - preoperative planning, with extensive and individualized information, education, and post-discharge arrangements,
  - screening for frailty and prior medical conditions, optimization of these conditions, and of anemia
  - Risk assessment for delirium and prophylaxis
  - Maintaining intraoperative body temperature
  - prompt return of personal sensory aids (glasses, walking aids, etc.)
  - early mobilization,
  - early removal of urinary catheters, and a proactive avoidance of postoperative nausea and vomiting, which could be a risk factor for aspiration

What is your response to the above recommendation on the Assessment of frailty or functional status?

1. Include unmodified
2. Include but modify
3. Include elsewhere in the protocol
4. Exclude

Citation

1. Byrnes A, Banks M, Mudge A, Young A, Bauer J. Enhanced Recovery After Surgery as an auditing framework for identifying improvements to perioperative nutrition care of older surgical patients. European journal of clinical nutrition. 2018 Jun;72(6):913-6.
2. Audisio RA. Tailoring surgery to elderly patients with cancer. Journal of British Surgery. 2016 Jan;103(2):e10-1.
3. Slieker J, Frauche P, Jurt J, Addor V, Blanc C, Demartines N, Hübner M. Enhanced recovery ERAS for elderly: a safe and beneficial pathway in colorectal surgery. International journal of colorectal disease. 2017 Feb;32:215-21.
4. Launay-Savary MV, Mathonnet M, Theissen A, Ostermann S, Raynaud-Simon A, Slim K. Are enhanced recovery programs in colorectal surgery feasible and useful in the elderly? A systematic review of the literature. Journal of Visceral Surgery. 2017 Feb 1;154(1):29-35.
5. Millan M. Enhanced recovery after surgery in elderly and high-risk patients. Annals of Laparoscopic and Endoscopic Surgery. 2020 Oct 20;5.
   - 1. Optimization (Smoking and alcohol cessation, HIV screening, Anemia screening)

Evidence box

***Smoking:*** *A higher risk of surgical complications is linked to smoking. Four to eight weeks of smoking cessation are required to lower respiratory and wound-healing complications.*

***Heavy alcohol consumption:*** *Alcohol suppresses the immune system during the perioperative phase and has a detrimental impact on the catabolic stress response. More than two units of alcohol per day were linked to a higher risk of postoperative infections in a recent systematic review and meta-analysis. It was also demonstrated in a 2012 Cochrane review and a follow-up sub-analysis that stopping alcohol use for at least four weeks was linked to fewer complications but had no effect on length of stay or mortality.*

***Anemia:*** *Preoperative anemia is associated with an increased risk of postoperative complications, increased rate of blood transfusion and mortality, and may worsen long-term oncology outcomes. Anemia is common in patients presenting for surgery with reported prevalence rates of up to 31.1%. Blood transfusion should be avoided as it has the potential for significant short- and long-term complications and is a scarce resource in most LMICs. Oral iron is inexpensive and easy to administer but may be poorly tolerated, especially in patients with gastrointestinal cancer. Intravenous iron has a low risk of adverse reactions and is more effective than oral iron at restoring hemoglobin concentrations in both iron deficiency anemia and anemia of chronic disease. A 2021 systematic review, which included 10 RCTs and 1039 participants, showed that preoperative IV iron supplementation decreased blood transfusion by 16% and was not associated with an increased incidence of any adverse effects across the groups.*

***HIV and Surgery***

*Following surgery, patients with HIV infection may experience higher morbidity. A viral load of over 10,000 copies/ml or an absolute CD4 count below 200 cells/cc3 are specific risk factors that impact surgical morbidity. Patients with low viral loads and CD4 counts above 200 cells/cc3 should be assessed based on predetermined preoperative criteria since their risk of postoperative problems is comparable to that of the general population. The prevalence of HIV in Ethiopia remains high in urban areas, where estimates indicate a rate of three percent. In areas of high prevalence voluntary universal screening test programmes should be encouraged*

Recommendation: Screening and optimization for smoking, heavy alcohol intake, anemia, and HIV is recommended.

- Smoking: Smoking cessation is recommended, preferably 4 weeks or more before the operation.
- Heavy alcohol: Preoperative abstinence of alcohol for 4 weeks before surgery is recommended.
- Anemia: Routine preoperative screening of Anemia is recommended. The type and cause of anemia should be investigated, and treated accordingly. Iron deficiency anemia should be treated with oral iron for patients who tolerate it. Intravenous Iron should be given to patients who need to be optimized urgently (cancer patients), and for patients who develop serious GI upset with oral Iron. Preoperative transfusion should be avoided when it is possible. Medical management of preoperative anemia takes time and should be planned at least 3–4 weeks before elective surgery.
- HIV: Routine screening for HIV/ AIDS, and CD4+ count and/or Viral load for HIV-positive patients is recommended. Optimize CD4+ count to >200/µl, and/or > 10,000 viral copies/ml before elective surgeries for non-urgent surgeries.

What is your response to the above recommendation on Optimization (Smoking and alcohol cessation, HIV screening, Anemia screening)?

1. Include unmodified
2. Include but modify
3. Include elsewhere in the protocol
4. Exclude

Citations

1. Oodit, Ravi, et al. "Guidelines for perioperative care in elective abdominal and pelvic surgery at primary and secondary hospitals in low–middle-income countries (LMIC’s): enhanced recovery after surgery (ERAS) society recommendation." *World Journal of Surgery* 46.8 (2022): 1826-1843.
2. Davison SP, Reisman NR, Pellegrino ED, Larson EE, Dermody M, Hutchison PJ. Perioperative guidelines for elective surgery in the human immunodeficiency virus–positive patient. Plastic and reconstructive surgery. 2008 May 1;121(5):1831-40.
3. Yilema SA, Shiferaw YA, Belay AT, Belay DB. Mapping the spatial disparities of HIV prevalence in Ethiopian zones using the generalized additive model. Scientific Reports. 2024 Mar 14;14(1):6215.
   - 1. Preoperative nutritional care

Evidence box

*Individuals who have unintentional weight loss of 5% of body mass over three months or 10% over six months are at higher risk for mortality, surgical complications, and poorer long-term oncology outcomes. Nutritional supplementation is linked to a decrease in anastomotic leakage and infectious complications in malnourished patients. Patients at increased risk should receive nutritional treatment preferably using the oral route for at least 7–14 days before surgery, even if surgery has to be delayed.*

Recommendations

- It is recommended to assess the nutritional status before surgery routinely
- Perioperative nutritional support therapy is indicated in patients with malnutrition and those at nutritional risk. Perioperative nutritional therapy should also be initiated if it is anticipated that the patient will be unable to eat for more than five days perioperatively.
- If the energy and nutrient requirements cannot be met by oral and enteral intake alone (<50% of caloric requirement) for more than seven days, a combination of enteral and parenteral nutrition (PN) is recommended. (when PN is accessible, and affordable)
- Preoperative fasting from midnight is unnecessary in most patients. Patients undergoing surgery, who are considered to have no specific risk of aspiration, shall drink clear fluids until 2 h before anesthesia. Solids shall be allowed until 6 h before anesthesia,
- To impact postoperative insulin resistance and length of stay (LOS), preoperative oral carbohydrate treatment can be considered in patients undergoing major surgery.

What is your response to the above recommendation on preoperative nutritional care?

1. Include unmodified
2. Include but modify
3. Include elsewhere in the protocol
4. Exclude

**Citation**

- - 1. Gustafsson UO, Scott MJ, Hubner M, Nygren J, Demartines N, Francis N, Rockall TA, Young-Fadok TM, Hill AG, Soop M, De Boer HD. Guidelines for perioperative care in elective colorectal surgery: Enhanced Recovery After Surgery (ERAS®) Society recommendations: 2018. World journal of surgery. 2019 Mar 15;43:659-95.
    2. Oodit, Ravi, et al. "Guidelines for perioperative care in elective abdominal and pelvic surgery at primary and secondary hospitals in low–middle-income countries (LMIC’s): enhanced recovery after surgery (ERAS) society recommendation." *World Journal of Surgery* 46.8 (2022): 1826-1843.
    3. Martínez-Ortega AJ, Piñar-Gutiérrez A, Serrano-Aguayo P, González-Navarro I, Remón-Ruíz PJ, Pereira-Cunill JL, García-Luna PP. Perioperative nutritional support: a review of current literature. Nutrients. 2022 Apr 12;14(8):1601.
    4. Weimann A, Braga M, Carli F, Higashiguchi T, Hübner M, Klek S, Laviano A, Ljungqvist O, Lobo DN, Martindale RG, Waitzberg D. ESPEN practical guideline: Clinical nutrition in surgery. Clinical Nutrition. 2021 Jul 1;40(7):4745-61.
    5. Bowel preparation

Evidence box

***mechanical bowel preparation****: is an oral preparation given before surgery to clear fecal material from the bowel lumen. There are a number of different preparations available including polyethylene glycol, mannitol, and sodium picosulphate. Rectal enemas may also be administered before low anterior resections to ensure that the rectum is empty.*

*Mechanical bowel preparation (MBP) has long been believed to lessen anastomotic leakage and surgical site infections. However, its use is linked to electrolyte imbalances, pre-operative dehydration, patient discontent, disturbed sleep the night before surgery, and elevated anxiety levels.*

*Overall, the use of MBP versus either no bowel preparation at all or a single rectal enema was not linked to a statistically significant difference in the incidence of anastomotic leak, surgical site infection, intra-abdominal collection, mortality, reoperation, or overall hospital length of stay, according to a meta-analysis of 23 randomized controlled trials and 13 observational studies. Once more, no discernible variation by preparation method was found in any clinical outcome measure when only evidence from randomized controlled trials was examined.*

*MBP has remained a dogmatic practice in colorectal surgeries. Nevertheless, numerous robust meta-analyses and RCT studies support the removal of standard MBP by showing that it is not beneficial for patients having elective colon and rectal surgeries.*

- ***Majority of the studies conclude by advising that mechanical bowel preparation before elective colorectal surgery can safely be abandoned.***

***Oral antibiotics bowel preparation (OABP):*** *Most studies show OABP is associated with reduced SSI rates, shorter LOS, and fewer readmissions.*

***Dietary restriction in the context of Colorectal surgeries****: There are no conclusive high-quality studies on outcomes of the practice of restricting diet to clear fluids or low residue diet a day before surgery in patients undergoing colorectal elective surgeries, despite popular practice.*

Recommendations:

- The use of mechanical bowel preparation in conjunction with systemic antibiotic prophylaxis is not recommended for routine colonic surgery due to the potential for dehydration and discomfort, but it may be utilized for rectal surgery.
- As per hospital procedure, parenteral antibiotic prophylaxis and the use of oral antibiotics for bowel preparation should continue.
- Currently, there is insufficient data to alter the practice of dietary restriction the day before scheduled elective colorectal procedures.

What is your response to the above recommendation on Bowel preparation?

1. Include unmodified
2. Include but modify
3. Include elsewhere in the protocol
4. Exclude

Citation

- - 1. Gustafsson UO, Scott MJ, Hubner M, Nygren J, Demartines N, Francis N, Rockall TA, Young-Fadok TM, Hill AG, Soop M, De Boer HD. Guidelines for perioperative care in elective colorectal surgery: Enhanced Recovery After Surgery (ERAS®) Society recommendations: 2018. World journal of surgery. 2019 Mar 15;43:659-95.
    2. Oodit, Ravi, et al. "Guidelines for perioperative care in elective abdominal and pelvic surgery at primary and secondary hospitals in low–middle-income countries (LMIC’s): enhanced recovery after surgery (ERAS) society recommendation." *World Journal of Surgery* 46.8 (2022): 1826-1843.
    3. Rollins KE, Javanmard-Emamghissi H, Lobo DN. Impact of mechanical bowel preparation in elective colorectal surgery: a meta-analysis. World journal of gastroenterology. 2018 Jan 1;24(4):519.
    4. Pineda CE, Shelton AA, Hernandez-Boussard T, Morton JM, Welton ML. Mechanical bowel preparation in intestinal surgery: a meta-analysis and review of the literature. Journal of gastrointestinal surgery. 2008 Nov 1;12(11):2037-44.
    5. Contant CM, Hop WC, van't Sant HP, Oostvogel HJ, Smeets HJ, Stassen LP, Neijenhuis PA, Idenburg FJ, Dijkhuis CM, Heres P, van Tets WF. Mechanical bowel preparation for elective colorectal surgery: a multicentre randomised trial. The Lancet. 2007 Dec 22;370(9605):2112-7.
    6. Morris MS, Graham LA, Chu DI, Cannon JA, Hawn MT. Oral antibiotic bowel preparation significantly reduces surgical site infection rates and readmission rates in elective colorectal surgery. Annals of surgery. 2015 Jun 1;261(6):1034-40.
    7. Preoperative fasting and CHO loading

Evidence box

*Fasting after midnight has become commonplace in elective surgery to prevent pulmonary aspiration, however, recent studies have shown no scientific evidence to support this practice. Clear fluids can be safely administered up to two hours and a light meal up to six hours prior to elective procedures requiring general anesthesia, regional anesthesia, or procedural sedation and analgesia in both adults and children, according to many RCTs.*

*Major abdominal surgery, especially open surgery, is associated with insulin resistance. This drives the catabolic response and results in an increased risk of complications. Preoperative administration of oral carbohydrates (complex CHO-maltodextrin, 12.5%, 285 mOsm/kg, 800 ml in the evening before surgery and 400 ml 2–3 h before induction of anesthesia) has been shown to attenuate the catabolic response induced by overnight fasting and surgery.*

*Patients undergoing emergency surgery or those with known delayed gastric emptying or gastrointestinal motility disorders may not be able to properly receive oral fluids, including CHOs 2-3 hours before surgery.*

Recommendation: Patients undergoing elective gastrointestinal and hepatobiliary surgeries should be permitted to consume clear fluids, such as CHO drinks, up to two hours prior to the start of anesthesia, and to eat full meals until eight hours and light meals until six hours. Emergency patients and those with delayed stomach emptying should fast for six hours prior to surgery or overnight.

What is your response to the above recommendation on Preoperative fasting and CHO loading?

1. Include unmodified
2. Include but modify
3. Include elsewhere in the protocol
4. Exclude

Citation

1. Gustafsson UO, Scott MJ, Hubner M, Nygren J, Demartines N, Francis N, Rockall TA, Young-Fadok TM, Hill AG, Soop M, De Boer HD. Guidelines for perioperative care in elective colorectal surgery: Enhanced Recovery After Surgery (ERAS®) Society recommendations: 2018. World journal of surgery. 2019 Mar 15;43:659-95.
2. Oodit, Ravi, et al. "Guidelines for perioperative care in elective abdominal and pelvic surgery at primary and secondary hospitals in low–middle-income countries (LMIC’s): enhanced recovery after surgery (ERAS) society recommendation." *World Journal of Surgery* 46.8 (2022): 1826-1843.
3. Ljungqvist O, Søreide E. Preoperative fasting. Journal of British Surgery. 2003 Apr;90(4):400-6.
   - 1. Preoperative fluid and electrolyte therapy

Evidence box

*Intravenous fluid therapy is required for most surgical patients, but inappropriate regimens are commonly prescribed. Patients must arrive at the anesthetic room in a state close to euvolemia, with any fluid and electrolyte imbalances corrected beforehand. When assessing fluid status, it is important to consider pre-existing comorbidities. To minimize preoperative fluid and electrolyte deficits, prolonged fasting should be avoided, and clear liquids, including carbohydrate drinks, can be provided up to 2 hours before anesthesia induction. Mechanical bowel preparation can lead to significant loss of total body water, potentially resulting in fluid and electrolyte imbalances, even if oral fluids are allowed. Dehydration and electrolyte imbalances can occur in older persons for various reasons, from iatrogenic factors such as polypharmacy and unmonitored diuretic use to physical limitations limiting fluid intake access. “Normal” saline (0.9% sodium chloride) is not physiological and can result in sodium overload and hyperchloremic acidosis. Some patients may need intravenous fluid therapy to address these deficits and enhance their outcomes, however, perioperative IV fluid regimen should be individualized.*

R

Recommendation: Fluid and electrolyte deficits or excesses should be corrected before patients are transported to the operating room. Preoperative prolonged fasting should be avoided.

What is your response to the above recommendation on Preoperative fluid and electrolyte therapy?

1. Include unmodified
2. Include but modify
3. Include elsewhere in the protocol
4. Exclude

Citation

1. Gustafsson UO, Scott MJ, Hubner M, Nygren J, Demartines N, Francis N, Rockall TA, Young-Fadok TM, Hill AG, Soop M, De Boer HD. Guidelines for perioperative care in elective colorectal surgery: Enhanced Recovery After Surgery (ERAS®) Society recommendations: 2018. World journal of surgery. 2019 Mar 15;43:659-95.
2. Myles PS, Andrews S, Nicholson J, Lobo DN, Mythen M. Contemporary approaches to perioperative IV fluid therapy. World journal of surgery. 2017 Oct;41:2457-63.
   - 1. PONV prophylaxis

Evidence box

*According to current estimates, the overall incidence of postoperative nausea and vomiting (PONV) is between 20 and 30 percent. In addition to causing patient discontent, PONV can lead to a longer hospital stay, a nasogastric tube being placed, delayed oral feeding, prolonged intravenous fluids, and higher medical expenses.
Patients are at risk for PONV due to a variety of risk factors. Apfel et al. developed the most popular scoring method, which focused on just four risk factors: female gender, a history of motion sickness or PONV, non-smoking status, and postoperative painkiller usage.
Antiemetics and complete intravenous anesthesia with propofol in place of inhalational drugs are part of the multimodal approach to PONV within an ERAS program. Nitrous oxide should also be avoided.
The occurrence of PONV may also be influenced by other variables, such as decreased preoperative fasting, carbohydrate loading, proper hydration, and increased inspired oxygen concentrations. The prevalence of PONV may also be indirectly impacted by the use of non-steroidal anti-inflammatory drugs (NSAIDs) and regional anesthesia techniques as opioid-sparing measures. Serotonin antagonists such as ondansetron 4 mg intravenously and dopamine antagonists administered intravenously at the end of surgery are excellent examples of antiemetic drugs. It's also been demonstrated that administering 4–5 mg intravenously of dexamethasone during anesthesia induction works well. Rescue therapy with an antiemetic of a different class should be used if PONV is present after surgery unless more than six hours have passed since the last antiemetic administration.*

Recommendation:

- All patients should be evaluated for a multimodal approach to PONV prevention.
- First-line antiemetics should ideally be used in a two- drug combination prophylactic for patients with one or two risk factors. Two to three antiemetics should be administered to patients who have at least two risk factors.
- Salvage therapy should be administered using a multimodal strategy with different classes of medications from those used for prophylaxis if nausea and/or vomiting persist after prophylaxis.

What is your response to the above recommendation on PONV prophylaxis?

1. Include unmodified
2. Include but modify
3. Include elsewhere in the protocol
4. Exclude

Citation

1. Oodit, Ravi, et al. "Guidelines for perioperative care in elective abdominal and pelvic surgery at primary and secondary hospitals in low–middle-income countries (LMIC’s): enhanced recovery after surgery (ERAS) society recommendation." *World Journal of Surgery* 46.8 (2022): 1826-1843.
2. Gustafsson UO, Scott MJ, Hubner M, Nygren J, Demartines N, Francis N, Rockall TA, Young-Fadok TM, Hill AG, Soop M, De Boer HD. Guidelines for perioperative care in elective colorectal surgery: Enhanced Recovery After Surgery (ERAS®) Society recommendations: 2018. World journal of surgery. 2019 Mar 15;43:659-95.
3. Feldheiser A, Aziz O, Baldini G, Cox BP, Fearon KC, Feldman LS, Gan TJ, Kennedy RH, Ljungqvist O, Lobo DN, Miller T. Enhanced Recovery After Surgery (ERAS) for gastrointestinal surgery, part 2: consensus statement for anaesthesia practice. Acta Anaesthesiologica Scandinavica. 2016 Mar;60(3):289-334.
   1. Specific Recommendations for Intra-operative Components

2.2.1. Surgical safety checklist

Evidence box

*Utilization of the surgical safety checklist (SSC) which was published by WHO in 2008 with 19 items and three pause points has been shown to reduce perioperative morbidity and mortality in patients undergoing surgery in LMIC/ HIC, as it dictates appropriate actions to be taken in the operating room and creating a common understanding between the operating team. Even though it has a significant effect on surgical outcomes, its applicability, and adoption in LMIC are very poor (20%-40%) due to multiple reasons. So, including SSC in the ERAS-Protocol may provide compliance on its utilization for improved patient safety and improved patient perioperative care in terms of reduction of morbidity and mortality.*

Recommendation**:**  SSC should be utilized properly and effectively as a routine practice. All the items should be filled in before the start of the surgery.

What is your response to the above recommendation on surgical safety checklist utilization?

1. Include unmodified
2. Include but modify
3. Include elsewhere in the protocol
4. Exclude

Citation

1. Ljungqvist O, Scott M, Fearon K (2017) Enhanced recovery after surgery. JAMA Surg 152(3):292
2. World Health Organization (2021) Implementation manual: WHO surgical safety checklist (first edition). <https://apps.who.int/iris/handle/10665/70046/>
3. Haynes A, Weiser T, Berry W et al (2009) A surgical safety checklist to reduce morbidity and mortality in a global population.N Engl J Med 360(5):491–499
4. Delisle M, Pradarelli J, Panda N et al (2020) Variation in global uptake of the surgical safety checklist. Br J Surg107(2):151–160

2.2.2. Antimicrobial prophylaxis

Evidence box

*According to many studies, meta-analysis and cochcrein review, providing prophylactic IV Antibiotics has demonstrated reduction in Surgical site infection (SSI) which is an infection occurring on the incision site or deep organ spaces within 30 days postoperative period. SSI occurs in a rate of 23.5% post laparotomy more of the infections occur in LMIC compared to HIC.*

*For gastrointestinal and hepatobiliary surgeries, the choice of antibiotics is first-generation cephalosporins as they have broad spectrum coverage and antibiotics covering anaerobes. Antibiotics should be provided intravenously 1 hr. before incision. For patients with penicillin/Cephalosporin sensitivity clindamycin and gentamycin or quinolones (Ciprofloxacin) are the alternatives.*

**Recommendations:** - Providing prophylactic IV antibiotics within 1 hour before skin incision is highly recommended.

What is your response to the above recommendation on antimicrobial prophylaxis?

1. Include unmodified
2. Include but modify
3. Include elsewhere in the protocol
4. Exclude

Citation

1. GlobalSurg Collaborative (2018) surgical site infection after gastrointestinal surgery in high-income, middle-income, and low-income countries: a prospective, international, multicenter cohort study. Lancet Infect Dis 18(5):516–525
2. Shrime M, Dare A, Alkire B et al (2015) Catastrophic expenditure to pay for surgery worldwide: a modelling study. Lancet Glob Health 3(Suppl 2(02)):S38-44
3. Nelson R, Gladman E, Barbateskovic M (2014) Antimicrobial prophylaxis for colorectal surgery. Cochrane Database of Syst Rev.https://doi.org/10.1002/14651858.CD001181.pub4
4. Weber W, Mujagic E, Zwahlen M et al (2017) Timing of surgical antimicrobial prophylaxis: a phase 3 randomized controlled trial. Lancet Infect Dis 17(6):605–614

**2.2.3.** Venous thromboembolism (VTE) prophylaxis: compression stocking and/or intermittent pneumatic compression together with either a LMWH or unfractionated heparin

Evidence box

*It is known that VTE has potentially fatal complications including pulmonary hypertension, Cardiac failure, and post-thrombotic syndrome.* *Malignancy, obesity, pelvic surgery, pre-operative immunosuppressant, immobility, and a hypercoagulable condition are established risk factors linked to an increased risk of VTE.*

*In recent eras, evidence has shown the benefit of VTE prophylaxis in patients undergoing Abdominal and Colorectal surgeries. As a result, the use of compression stocking and/or intermittent pneumatic compression together with either an LMWH or unfractionated heparin should be administered during the hospitalization period, as opposed to older recommendations for a 28-day post-operative period.*

*Previous ERAS recommendations and other guidelines (NICE, NHMRC) advised ETP (28 days) for patients after major abdominal or pelvic cancer surgery, based on a Cochrane meta-analysis of four RCTs. Current evidence has shown that the incidence of post-discharge VTE (0.60–0.73%), DVT (0.29–0.48%), and PE (0.26–0.40%) is very low.*

*The value of extended prophylaxis for elective procedures performed on low-risk patients in tertiary hospitals in LMICs must be weighed against practical issues (such as cost, availability, transport, and storage).*

Recommendations: We recommend risk-stratifying patients for VTE using risk assessment tools such as the Caprini model. Routine VTE prophylaxis is recommended while patients are in the hospital. High-risk patients should get combined prophylaxis (LMWH and Mechanical), and moderate-risk patients should get either LMWH or Mechanical, i.e. intermittent pneumatic compressor (IPC). Low-risk patients can be treated with mechanical prophylaxis, i.e. either IPC or Elastic Stockings (ES) depending on the availability.

What is your response to the above recommendation on VTE prophylaxis?

1. Include unmodified
2. Include but modify
3. Include elsewhere in the protocol
4. Exclude

Citation

1. Gee E (2019) The National VTE Exemplar Centres Network response to implementation of updated NICE guidance: venous thromboembolism in over 16s: reducing the risk of hospitalacquired deep vein thrombosis or pulmonary embolism (NG89). Br J Haematol 186(5):792–793
2. Kakkos S, Caprini J, Geroulakos G et al (2016) Combined intermittent pneumatic leg compression and pharmacological prophylaxis for prevention of venous thromboembolism. Cochrane Database of Syst Rev. <https://doi.org/10.1002/> 14651858.CD005258.pub3
3. Rausa E, Kelly M, Asti E et al (2018) Extended versus conventional thromboprophylaxis after major abdominal and pelvicurgery: systematic review and meta-analysis of randomized clinical trials. Surgery 164(6):1234–1240
4. Iannuzzi JC, Aquina CT, Rickles AS et al (2016) Risk factorsfor postdischarge venothromboembolism after colorectal resection.Dis Colon Rectum 59:224–229

2.2.4. Standard anesthesia protocol: Short-acting anesthetic agents, lung-protective ventilation, and complete reversal of neuromuscular blockade

Evidence box

*The ultimate goal of anesthesia in surgery is to provide adequate amnesia, hypnosis, muscle relaxation, analgesia, in assurance of adequate circulation and oxygenation with limited or no residual side effects or complications which helps for rapid mobilization and recovery.*

*In order to achieve early recovery, mobilization, and oral intake initiation, using short-acting anesthetic agents, and lung-protective ventilation, achieving complete reversal of neuromuscular blockade is effective.*

*Because it has a rapid onset of action and recovery with reduced nausea and vomiting propofol is being used as an induction medication. Even if there is no evidence supporting it, anesthesia is maintained via volatile anesthesia (e.g. Isoflurine/ sevoflurine) and/or total intravenous anesthesia (e.g. dexmedetomidine, ketamine).* *Additionally, dexmedetomidine lessens the need for opioids, and ketamine may lessen persistent postoperative pain.*

Recommendations**:** - It is advised to use a conventional anesthetic regimen that includes short-acting anesthetics, cerebral monitoring to enhance recovery and lower the risk of postoperative delirium, and monitoring of the degree and total reversal of neuromuscular block.

Before extubating, the neuromuscular block must be completely reversed.

What is your response to the above recommendation on Standard anesthesia protocol?

1. Include unmodified
2. Include but modify
3. Include elsewhere in the protocol
4. Exclude

Citation

1. Buhre W, Disma N, Hendrickx J et al (2019) European society of anaesthesiology task force on nitrous oxide: a narrative review of its role in clinical practice. Br J Anaesth 122(5):587–604
2. Naguib M, Brull SJ, Kopman AF et al (2018) consensus statement on perioperative use of neuromuscular monitoring. Anesth Analg 127(1):71–80
3. Odor PM, Bampoe S, Gilhooly D et al (2020) Perioperative interventions for prevention of postoperative pulmonary complications: systematic review and meta-analysis. BMJ 368:m540

2.2.5. Normothermia: Active Vs. Passive warming

Evidence box

*Both neuraxial and general anesthesia interfere with shivering and vasoconstriction, which causes the temperature to shift from the core to the periphery and results in more heat loss than heat production.* *Even minor unintentional perioperative hypothermia (IPH) has been linked to negative outcomes; in one meta-analysis, blood loss rose by 16% and the rate of blood transfusions increased by 22% at a median temperature of 35.6 °C. It also increases the risk of surgical site infections, prolonged postoperative acute care units (PACU), and hospital stays.*

*In the first half hour following the onset of general or neuro-axial anesthesia, the patient's core temperature decreases by 0.5 to 1.5 degrees.* *For all patients undergoing procedures longer than thirty minutes, active warming should be performed. A number of techniques are employed to preserve normothermia, such as the use of forced air blankets, heated intravenous fluid, anesthetic gases, and underbody warming mattresses. Before starting active warming, the patient should be exposed to an operating room temperature of at least 21 °C.*

*It is widely acknowledged that sustaining normothermia—a temperature of 36 °C or higher—is crucial for patients having major surgery, including colon surgery.*

**Recommendations: -** Intraoperative and postoperative maintenance of normal body temperature >36*°C* is mandatory. This can be achieved by having accurate core temperature measurements. Warming blankets, warm IV fluids, blood components, and radiant warmers can be used for maintaining intraoperative normothermia in our setting.

What is your response to the above recommendation on maintaining intraoperative normotermia?

1. Include unmodified
2. Include but modify
3. Include elsewhere in the protocol
4. Exclude

Citation

1. Billeter AT, Hohmann SF, Druen D et al (2014) Unintentional perioperative hypothermia is associated with severe complications and high mortality in elective operations. Surgery 156:1245–1252
2. Sessler DI (2016) Perioperative thermoregulation and heat balance. Lancet 387:2655 2664
3. Rajagopalan S, Mascha E, Na J et al (2008) the effects of mild perioperative hypothermia on blood loss and transfusion requirement. Anesthesiology 108:71–77
4. Riley C, Andrzejowski J (2018) inadvertent perioperative hypothermia. BJA Educ 18(8):227–233

2.2.6. Multimodal opioid-sparing analgesia

Evidence box

*Early mobilization and recovery of bowel function are facilitated by a multimodal analgesia approach that spares opioids.*

*During surgery, short-acting opioid analgesics (such as remifentanil) consistently promote a quick recovery, although there is worry that they could cause hyperalgesia. Ketamine, magnesium, the steroids dexmedetomidine, lidocaine infusions, and gabapentinoids are additional substances to take into account for analgesia during surgery. Few studies have compared the analgesic benefits and adverse effects to those of other medications.*

*For open abdominal surgery, a mid-thoracic epidural (TEA), T7-10, is advised. It needs to be started before surgery and continued during the procedure, ideally for 48 to 72 hours after the procedure. Several RCTs have demonstrated that TEA is superior to systemic opioids for open abdominal surgery. Reductions in insulin resistance, protein loss, the surgical catabolic response, and the recovery period for bowel function are other advantages. TEA's disadvantages include failure rates of up to 33.6% and hypotension with a danger of fluid overload and urine retention.* *It has been demonstrated that spinal analgesia works well in an ERAS care pathway for MIS colorectal surgery. A local anesthetic and a long-acting opioid are frequently combined. A 1.5–2 ml total volume is advised to prevent a high spinal block. Delay in respiratory depression is a significant problem when using spinal morphine, particularly in older patients. After receiving spinal morphine, patients must be cautiously watched during the first twenty-four hours.*

*During the perioperative phase, intravenous lidocaine infusion reduces the need for intraoperative anesthetics, pain scores, postoperative analgesics, the length of hospital stay, and the recovery of bowel function.*

*Transversus abdominal plane (TAP), subcostal, and rectus blocks are examples of abdominal wall blocks. While subcostal and rectus block work well as adjuncts to offer a block for the upper abdomen, TAP blocks provide analgesia from T10 to L1, or below the umbilicus. According to recent systematic studies, TAP blocks have been associated with shorter lengths of stay (LOS), earlier recovery of bowel function, and decreased opioid intake in abdominal and gynecologic surgeries.*

*As part of a multimodal regimen, paracetamol and non-steroidal anti-inflammatory medicines (NSAIDs) are essential medications during the perioperative phase. Opioids should be avoided, though, if necessary, short-acting opioids should be utilized. Both oral and intravenous forms of paracetamol are accessible, reasonably priced, and have few adverse effects.*

Recommendations:

- TEA analgesia is recommended in open abdominal surgeries, if the epidural catheter, set, and expertise are affordable and accessible.
- Spinal analgesia is recommended in laparoscopic surgeries.
- Abdominal wall blocks can be used when TEA is not possible.
- The combination of paracetamol and NSAIDS is recommended as baseline multimodal analgesics in the postoperative period unless specific contraindications exist.

What is your response to the above recommendation on multimodal opioid-sparing analgesia?

1. Include unmodified
2. Include but modify
3. Include elsewhere in the protocol
4. Exclude

Citation

1. Bell R, Dahl J, Moore R et al (2015) Perioperative ketamine for acute postoperative pain. Acta Anaesthesiol Sacnd 49(10):1205–1228
2. Kaye A, Chernobylsky D, Thakur P et al (2020) Dexmedetomidine in enhanced recovery after surgery (ERAS) protocols for postoperative pain. Curr Pain Headache Rep 24(5):21
3. Weibel S, Jelting Y, Pace N et al (2018) Continuous intravenous perioperative lidocaine infusion for postoperative pain and recovery in adults. Cochrane Database Syst Rev 6(6). https://doi.org/10.1002/14651858.CD009642
4. Hughes M, Ventham N, McNally S et al (2014) Analgesia after open abdominal surgery in the setting of enhanced recovery surgery. JAMA Surg 149(12):1224
5. Block B, Liu S, Rowlingson A et al (2003) Efficacy of postoperative epidural analgesia. JAMA 290(18):2455
6. Werawatganon T, Charuluxananan S (2013) Patient controlled nintravenous opioid analgesia versus continuous epidural analgesia for pain after intra-abdominal surgery. Cochrane Library.https://doi.org/10.1002/14651858.CD004088
7. Po¨pping D, Elia N, Van Aken H et al (2014) Impact of epidural analgesia on mortality and morbidity after surgery. Ann Surg259(6):1056–1067
8. Baker H, Yates V, Addison D et al (2015) The effect of anaesthetist grade and frequency of insertion on epidural failure:a service evaluation in a United Kingdom teaching hospital. BMC Anesthesiol. 15(1):5–5. https://doi.org/10.1186/1471-2253-15-5
9. Holte K, Foss N, Svense´n C, Lund et al (2004) Epidural anesthesia, hypotension, and changes in intravascular volume. Anesthesiology. 100(2):281–286
10. Joshi G, Bonnet F, Kehlet H (2013) Evidence-based postoperative pain management after laparoscopic colorectal surgery. IntJ Colorectal Dis 15(2):146–155
11. Hu¨bner M, Blanc C, Roulin D et al (2015) Randomized clinical trial on epidural versus patient-controlled analgesia for laparoscopic colorectal surgery within an enhanced recovery pathway. Ann Surg 261(4):648–653 Peltrini R, Cantoni V, Green R et al (2020) Efficacy of transversus abdominis plane (TAP) block in colorectal surgery a systematic review and meta-analysis. Tech Colproctol 24(8):787–802
12. Hamid H, Ahmed A, Alhamo M et al (2021) Efficacy and safety profile of rectus sheath block in adult laparoscopic surgery: a meta-analysis. J Surg Res 261:10–17

2.2.7. Fluid balance: Goal-directed fluid therapy (GDFT)

Evidence box

*Maintaining intravascular volume is the primary goal of intravenous fluids (IVF) to prevent electrolyte imbalances and guarantee proper tissue and organ perfusion. Since difficulties can arise from both excessive and insufficient hydration, there is a limited range of ideal fluid management. Most of the time, skipping bowel preparation and consuming the recommended amount of fluids before surgery will guarantee that the patient is well-hydrated when they arrive at the hospital. The majority of patients will need balanced crystalloids (such as Ringer's lactate) during surgery. Because of the possibility of salt and fluid excess, 0.9% saline should not be used.*

Recommendations

- Near zero fluid balance is recommended.
- Fluid excess leading to a perioperative weight gain of more than 2.5 kg should be avoided, and a perioperative near-zero fluid balance approach should be preferred.
- Inotropes should be considered in patients with poor contractility CI < 2.5 l/min). 0.9% saline and saline-based solutions should be avoided, with balanced solutions preferred.
- In patients receiving epidural analgesia, arterial hypotension should be treated with vasopressors after ensuring the patient is normovolaemic.
- In the absence of surgical losses, the postoperative intravenous fluid should be discontinued, and oral intake (1.5 l/day) encouraged.

What is your response to the above recommendation on Fluid balance: Goal-directed fluid therapy (GDFT)?

1. Include unmodified
2. Include but modify
3. Include elsewhere in the protocol
4. Exclude

Citation

1. Thiele R, Raghunathan K, Brudney C (2016) American society for enhanced recovery (ASER) and perioperative quality initiative (POQI) joint consensus statement on perioperative fluid management within an enhanced recovery pathway for colorectal surgery. Perioper Med (Lond) 5(1):24
2. Egal M, de Geus H, van Bommel J et al (2016) Targeting oliguria reversal in perioperative restrictive fluid management does not influence the occurrence of renal dysfunction. Eur JAnaesthesiol 33(6):425–435
3. Padhi S, Bullock I, Li L et al (2013) Intravenous fluid therapy for adults in hospital: summary of NICE guidance. BMJ 347:7073–7073
4. Varadhan K, Lobo D (2010) A meta-analysis of randomized controlled trials of intravenous fluid therapy in major elective open abdominal surgery: getting the balance right. Proc Nutr Soc 69(4):488–498
5. Feldheiser A, Aziz O, Baldini G, Cox BP, Fearon KC, Feldman LS, Gan TJ, Kennedy RH, Ljungqvist O, Lobo DN, Miller T. Enhanced Recovery After Surgery (ERAS) for gastrointestinal surgery, part 2: consensus statement for anaesthesia practice. Acta Anaesthesiologica Scandinavica. 2016 Mar;60(3):289-334.
   1. Specific Recommendations for Post-operative Components
      1. Postoperative fluid and electrolyte therapy

Evidence box

*The key aim of intravenous fluid (IVF) is to maintain intravascular volume to ensure adequate tissue and organ perfusion and to avoid electrolyte imbalance. Postoperative fluid should be minimized to maintain normovolaemia and avoid fluid excess. The enteral route should be used in preference and the drip taken down at the earliest opportunity, not more than the morning after surgery. If IVF needs to be continued postoperatively a hypotonic crystalloid with 70-100 mmol/day of sodium and up to 1 mmol/kg/day of potassium should be used.*

*Any ongoing losses (e.g. diarrhea, vomiting) should be replaced with a balanced solution (e.g. ringer lactate), whereas as 0.9% saline solution should be avoided.*

Recommendation: Balanced crystalloids should be used over 0.9% saline solution. If the patient is normovolaemic, vasopressors should be used for postoperative management of epidural-induced hypotension. Patients should be encouraged to drink when fully recovered and offered an oral diet within 4 hours after abdominal surgery.

What is your response to the above recommendation on Postoperative fluid and electrolyte therapy?

1. Include unmodified
2. Include but modify
3. Include elsewhere in the protocol
4. Exclude

Citation

1. Gustafsson, U.O., Scott, M.J., Schwenk, W., Demartines, N., Roulin, D., Francis, N., McNaught, C.E., Macfie, J., Liberman, A.S., Soop, M. and Hill, A., 2013. Guidelines for perioperative care in elective colonic surgery: Enhanced Recovery After Surgery (ERAS®) Society recommendations. *World journal of surgery*, *37*, pp.259-284.
2. Oodit, R., Biccard, B.M., Panieri, E., Alvarez, A.O., Sioson, M.R., Maswime, S., Thomas, V., Kluyts, H.L., Peden, C.J., de Boer, H.D. and Brindle, M., 2022. Guidelines for perioperative care in elective abdominal and pelvic surgery at primary and secondary hospitals in low–middle-income countries (LMIC’s): enhanced recovery after surgery (ERAS) society recommendation. *World Journal of Surgery*, *46*(8), pp.1826-1843.
   - 1. Postoperative nutritional care

Evidence box

*Patients who have been kept fasting traditionally until the return of signs of bowel function is associated with an increased risk of postoperative infectious complications and delayed recovery. Early oral feeding (fluid immediately after surgery and solid after 4 hours), combined with measures to reduce postoperative ileus is associated with early return of bowel function, shorter hospital stays, and no increased risk of anastomotic leak.*

*RCTs of early oral feeding vs ‘nil by os’ show that early feeding reduces the risk of infection and hospital stay, whereas the risk of vomiting increased especially in the absence of multimodal anti-ileus therapy.*

Recommendation: For standard ERAS patients preoperative fasting should be minimized and postoperatively patients should be encouraged to take normal food as soon as possible after surgery.

- With moderate evidence early oral feeding is strongly recommended.

What is your response to the above recommendation on Postoperative nutritional care?

1. Include unmodified
2. Include but modify
3. Include elsewhere in the protocol
4. Exclude

Reference

1. Oodit, R., Biccard, B.M., Panieri, E., Alvarez, A.O., Sioson, M.R., Maswime, S., Thomas, V., Kluyts, H.L., Peden, C.J., de Boer, H.D. and Brindle, M., 2022. Guidelines for perioperative care in elective abdominal and pelvic surgery at primary and secondary hospitals in low–middle-income countries (LMIC’s): enhanced recovery after surgery (ERAS) society recommendation. *World Journal of Surgery*, *46*(8), pp.1826-1843.
2. Guidelines for perioperative care in elective colonic surgery: Enhanced Recovery After Surgery (ERAS^®^) Society recommendations Gustafsson, U.O. et al. Clinical Nutrition, Volume 31, Issue 6, 783 – 800
   - 1. Early structured mobilization plan

Evidence box

*Early mobilization is an essential component of perioperative care to improve recovery after surgery. The current recommendation is for the patient to sit out of bed for 30 minutes on day 0 and 6hr/day thereafter and start walking on day 1. Failure to mobilize on the 1^st^ postoperative day was one of the most common reasons for ERAS deviation and was associated with prolonged length of hospital stay.*

Patients and families should be educated preoperatively on the goals.

Recommendation: Even though available RCTs do not support the direct clinical beneficial effect of postoperative mobilization. Prolonged immobilization increases the risk of pneumonia, insulin resistance, and muscle weakness. Patients therefore should be mobilized as early as they tolerate.

What is your response to the above recommendation on an Early structured mobilization plan?

1. Include unmodified
2. Include but modify
3. Include elsewhere in the protocol
4. Exclude

Citation

1. Guidelines for perioperative care in elective colonic surgery: Enhanced Recovery After Surgery (ERAS^®^) Society recommendations Gustafsson, U.O. et al. Clinical Nutrition, Volume 31, Issue 6, 783 – 800
2. Smart, N.J. ∙ White, P. ∙ Allison, A.S. ...Deviation and failure of Enhanced Recovery After Surgery (ERAS) following laparoscopic colorectal surgery: early prediction model *Colorectal Dis.* 2012; 14:e727-e734
3. Oodit, R., Biccard, B.M., Panieri, E., Alvarez, A.O., Sioson, M.R., Maswime, S., Thomas, V., Kluyts, H.L., Peden, C.J., de Boer, H.D. and Brindle, M., 2022. Guidelines for perioperative care in elective abdominal and pelvic surgery at primary and secondary hospitals in low–middle-income countries (LMIC’s): enhanced recovery after surgery (ERAS) society recommendation. *World Journal of Surgery*, *46*(8), pp.1826-1843.
   - 1. Postoperative analgesia

Evidence box

*The optimal analgesia for major surgery should provide good pain relief; and allow early mobilization, early return of gut function, and oral feeding. The cornerstone of analgesia remains multimodal analgesia combining regional or local anesthetics and trying to avoid parenteral opioids.*

*The multimodal analgesia approach facilitates early mobilization and return of bowel function. Mid-thoracic epidural analgesia (TEA), T7-10, is recommended for open abdominal surgery. It should be started preoperatively continued intraoperative and postoperatively, 48-72h. TEA has been shown to be better than that of systemic opioid analgesia. Paracetamol and non-steroidal anti-inflammatory drugs are the key drugs in the perioperative period as part of multimodal analgesia.*

Recommendation: The combination of paracetamol and non-steroidal anti-inflammatories is recommended as baseline multimodal analgesics unless a specific contraindication exists.

TEA is recommended in open abdominal surgery

What is your response to the above recommendation on Postoperative analgesia?

1. Include unmodified
2. Include but modify
3. Include elsewhere in the protocol
4. Exclude

Citation

- - - 1. Oodit, R., Biccard, B.M., Panieri, E., Alvarez, A.O., Sioson, M.R., Maswime, S., Thomas, V., Kluyts, H.L., Peden, C.J., de Boer, H.D. and Brindle, M., 2022. Guidelines for perioperative care in elective abdominal and pelvic surgery at primary and secondary hospitals in low–middle-income countries (LMIC’s): enhanced recovery after surgery (ERAS) society recommendation. *World Journal of Surgery*, *46*(8), pp.1826-1843.
    1. Plan for opioid minimization

Evidence box

*An opioid sparing analgesia facilitates early return of bowel function and mobilization; short acting opioids (e.g. remifentanil) should be used if necessary. Opioids are best reserved for breakthrough pain. Other analgesic adjuncts such as gabapentinoids, magnesium and ketamine are being used.*

*The ability to provide the most effective opioid sparing analgesia that is safe and allows early mobilization and oral feeding, remains the ideal approach. This avoids the unwanted side effect of sedation, nausea and vomiting, delirium and gut dysfunction.*

Recommendation: Avoid or minimize opioids and apply multimodal analgesia in combination with epidural analgesia or TAP blocks when indicated.

- Multimodal opioid-sparing analgesia

What is your response to the above recommendation on the Plan for opioid minimization?

1. Include unmodified
2. Include but modify
3. Include elsewhere in the protocol
4. Exclude

Citation

1. Oodit, R., Biccard, B.M., Panieri, E., Alvarez, A.O., Sioson, M.R., Maswime, S., Thomas, V., Kluyts, H.L., Peden, C.J., de Boer, H.D. and Brindle, M., 2022. Guidelines for perioperative care in elective abdominal and pelvic surgery at primary and secondary hospitals in low–middle-income countries (LMIC’s): enhanced recovery after surgery (ERAS) society recommendation. *World Journal of Surgery*, *46*(8), pp.1826-1843.
   - 1. Prevention of postoperative ileus

Evidence box

*Postoperative ileus is the major cause of prolonged hospital stay and the key objective of enhanced recovery protocols. No prokinetic agent has been shown to be effective in attenuating or treating postoperative ileus. Mid-thoracic epidural analgesia is highly effective in preventing postoperative ileus, compared to IV opioids. Fluid overloading during and after surgery impairs GI function and should be avoided. Avoidance of NG decompression may reduce postoperative ileus. Postoperative use of chewing gum has a positive effect on postoperative duration of ileus. Oral magnesium and alvimopan (when opioid analgesia was used) showed improved bowel function without significant side effects in some RCTs.*

Recommendation: Mid-thoracic epidural analgesia should be utilized in open abdominal surgery. Fluid overload and NG decompression should be avoided. Chewing gums can be recommended. Provided that they are available, Oral Magnesium and alvimopan can be used when opioid analgesia is used.

What is your response to the above recommendation on the Prevention of postoperative ileus?

1. Include unmodified
2. Include but modify
3. Include elsewhere in the protocol
4. Exclude

Citations

1. Guidelines for perioperative care in elective colonic surgery: Enhanced Recovery After Surgery (ERAS^®^) Society recommendations Gustafsson, U.O. et al. Clinical Nutrition, Volume 31, Issue 6, 783 – 800
2. Xie, Gui-Sheng MD^a^; Ma, Liang MD^b^; Zhong, Jian-Hong MD^b,c,d,*^. Recovery of gastrointestinal functional after surgery for abdominal tumors: A narrative review. Medicine 103(44):p e40418, November 01, 2024. | DOI: 10.1097/MD.0000000000040418
   - 1. Standardized Criteria for Discharge

Evidence box

*Starting in the outpatient clinic beginning with preoperative education, the patient and family are provided with a daily plan for each day of hospitalization. These include milestones to achieve for diet and activities, information about the management of pain and drain, and the target discharge date if everything goes well. These are consistent with nursing protocols and clinical orders. Large posters on the ward can be used to reinforce the information. Patients, families, and nurses are also aware of discharge criteria. These address concerns that enhanced recovery programs are primarily tools to reduce hospital stays as milestones for recovery need to be met.*

*Discharge criteria: passing gas or stool, no fever, minimal pain (4/10), walking unattended, and eating.*

*Patients, families, or caregivers also receive written information that includes instructions, symptoms to monitor for that need to be reported, who to report them to, emergency contact information, and strategies to aid recovery.*

Recommendation: Using discharge criteria addresses concerns that enhanced recovery programs are primarily tools to reduce hospital stays as milestones for recovery need to be met.

The date of discharge should be individualized for the type of surgery and needs to be more objectively evaluated rather than using the length of hospital stay alone. Patients should be passing gas or stool, afebrile, with normal vital signs including minimal pain (4/10), walking unattended, and eating normally before discharge.

What is your response to the above recommendation on standardized criteria for discharge?

1. Include unmodified
2. Include but modify
3. Include elsewhere in the protocol
4. Exclude

Citations

1. Maessen, J.M.C., Dejong, C.H.C., Kessels, A.G.H., von Meyenfeldt, M. and Enhanced Recovery After Surgery (ERAS) Group, 2008. Length of stay: an inappropriate readout of the success of enhanced recovery programs. *World journal of surgery*, *32*, pp.971-975.
2. Jeong, O., Ryu, S.Y. and Park, Y.K., 2016. Postoperative functional recovery after gastrectomy in patients undergoing enhanced recovery after surgery: a prospective assessment using standard discharge criteria. *Medicine*, *95*(14), p.e3140.

**Section 3: Nobel Elements**

In section 3, you will be provided with the list of new elements that could be beneficial to the implementation of ERAS protocol in elective GI and HPB surgeries at Ethiopian tertiary hospital setups. These items were found based on your recommendations during the initial round of the ERAS protocol adaption survey. On a 5- 5-point Likert scale, ranging from 1- Must exclude to 5- Must include, please rate the recommendations in order to come to a consensus on whether to include the following components in the final protocol. You will be prompted to add any more remarks in the space that follows here.

- 1. Which of these New items do you think should be included in an ERAS Protocol adapted for elective GI and HPB surgery patients at tertiary hospitals in Ethiopia?

| N0. | Intervention | Must include | Should include | Neutral | Should exclude | Must exclude |
| --- | --- | --- | --- | --- | --- | --- |
| 1. | Assessment of social support and consideration of social determinants |  |  |  |  |  |
| 2. | Preoperative preparation of OJ patients ( fluid and electrolytes, Vit-K, antibiotics) |  |  |  |  |  |
| 3. | Preoperative respiratory exercise |  |  |  |  |  |
| 4. | Hospital ERAS team |  |  |  |  |  |
| 5. | Transfusion guideline and strategies to minimize blood loss |  |  |  |  |  |
| 6. | Hospital-to-home transition: Case manager |  |  |  |  |  |
| 7. | Continuous medical education (CME) in ERAS |  |  |  |  |  |
| 8. | Audit |  |  |  |  |  |

- 1. Please provide any suggestions for modification or additions

**Additional recommendations on comprehensive perioperative period pain management**

Evidence box

Postoperative pain is among the most frequently reported concerns by patients following surgery. Numerous studies have identified preoperative pain and anxiety as significant predictors of postoperative pain, both of which are positively associated with increased pain intensity after surgery. (Coppes, O.J.M.,*et al.* Patient and Surgery-Related Predictors of Acute Postoperative Pain. *Curr Pain Headache Rep* 24, 12 (2020) Mehari *et al.* (2022). Prevalence and Factors Associated with Acute Postoperative Pain after Emergency Abdominal Surgery. *The Open Pain Journal*. 15. 10.2174/18763863-v15-e2208250. Effective perioperative pain management is fundamental to enhanced recovery after surgery. Managing postoperative pain is complex and demands a collaborative, interprofessional approach that starts before surgery and extends throughout the recovery period. Conducting a comprehensive preoperative assessment helps identify patient-specific risk factors that could affect the intensity of postoperative pain, allowing for the development of personalized pain management strategies. Therefore, patient education and counseling, along with the use of preoperative analgesic and anxiolytic medications, should be specifically addressed during the preoperative assessment to optimize postoperative pain management (Darville-Beneby R, *et al*. The Impact of Preoperative Patient Education on Postoperative Pain, Opioid Use, and Psychological Outcomes: A Narrative Review. *Can J Pain*. 2023 Nov 28;7(2):2266751).

Although opioids remain a key component of acute pain management, their routine use in the perioperative period is increasingly limited due to adverse effects particularly gastrointestinal issues that slow gut motility and delay oral intake. Evidence shows that opioid-based anesthesia offers no advantage in postoperative pain relief and is associated with increased rates of nausea and vomiting compared to opioid-free approaches (Frauenknecht J, Kirkham KR, Jacot‐Guillarmod A, Albrecht E. Analgesic impact of intra‐operative opioids vs. opioid‐free anaesthesia: a systematic review and meta‐analysis. *Anaesthesia*. 2019 May;74(5):651-62). These complications, including ileus, can significantly hinder enhanced recovery efforts. As a result, minimizing opioid use through multimodal analgesia during the perioperative period is a key strategy to promote faster recovery and improve patient outcomes. (Nimmo SM, Foo IT, Paterson HM. Enhanced recovery after surgery: pain management. *Journal of surgical oncology.* 2017 Oct;116(5):583-91). This multimodal analgesia approach commonly includes the use of systemic non-opioid analgesics such as paracetamol, NSAIDs/COX-2 inhibitors, glucocorticoids, gabapentinoids, or their combinations to improve pain control while minimizing opioid use and its associated side effects.

Recommendation:

- All patients undergo a thorough preoperative assessment to identify individual risk factors that may influence postoperative pain severity, enabling the creation of tailored pain management plans.
- Patient education and counseling, as well as the use of preoperative analgesics and anxiolytics, be integral components of the preoperative assessment to enhance postoperative pain control.
- Perioperative pain management include paracetamol in combination with NSAIDs or COX-2 selective inhibitors, unless contraindicated initiated preoperatively or intraoperatively and continued for up to 72 hours after surgery.
  - NSAIDs should be used with caution in patients with a history of gastrointestinal bleeding, cardiovascular disease, or chronic renal disease due to the risk of adverse effects.
  - For patients at high risk of gastrointestinal complications, COX-2 selective inhibitors are recommended as safer alternatives to non-selective NSAIDs to reduce gastrointestinal toxicity.
  - Ketorolac does not increase the risk of perioperative bleeding and can be safely used for postoperative pain management when appropriate.
  - Avoid NSAIDs and COX-2 selective inhibitors after colonic or rectal anastomosis due to the increased risk of anastomotic leakage.
  - Assess for hepatotoxicity risk factors such as liver disease, advanced age, malnutrition, and intraoperative liver ischemia before prescribing paracetamol, and adjust the dose if necessary
  - Paracetamol is recommended as a basic analgesic even after liver resection, given its low risk of hepatotoxicity.
  - Administer a single low dose of IV dexamethasone intraoperatively for antiemetic effect; doses >0.1 mg/kg may be used as an effective adjunct to reduce postoperative pain and opioid consumption within a multimodal analgesia strategy.
- Gabapentinoids may be used pre-operative and intra-operative period as adjuncts when paracetamol or NSAIDs/COX-2 inhibitors are contraindicated. They reduce pain, opioid use, and nausea but should not be used routinely due to risks like dizziness, cognitive impairment, and respiratory depression, particularly when combined with opioids.
- Opioids should be reserved for rescue analgesia in the postoperative period and used only when other pain management options are inadequate, due to their potential for significant adverse effects

Citation

Molla YD, Alemu HT. The Role of Gabapentin in Enhanced Recovery After Surgery (ERAS) for Patients Undergoing Abdominal Procedures, A Systematic Review and Meta-Analysis. *Health Sci Rep.* 2025 Apr 29;8(5):e70813.

Patel AS, Abrecht CR, Urman RD. Gabapentinoid use in perioperative care and current controversies. *Current Pain and Headache Reports*. 2022 Feb;26(2):139-44.

The PROSPECT (PROcedure-SPECific postoperative pain managemenT) <https://esraeurope.org/prospect/>

Frauenknecht J, Kirkham KR, Jacot‐Guillarmod A, Albrecht E. Analgesic impact of intra‐operative opioids vs. opioid‐free anaesthesia: a systematic review and meta‐analysis. *Anaesthesia*. 2019 May;74(5):651-62).

Nimmo SM, Foo IT, Paterson HM. Enhanced recovery after surgery: pain management. *Journal of surgical oncology.* 2017 Oct;116(5):583-91

Coppes, O.J.M.,*et al.* Patient and Surgery-Related Predictors of Acute Postoperative Pain. *Curr Pain Headache Rep* 24, 12 (2020) Mehari *et al.* (2022).

Prevalence and Factors Associated with Acute Postoperative Pain after Emergency Abdominal Surgery. *The Open Pain Journal*. 15. 10.2174/18763863-v15-e2208250

Darville-Beneby R, *et al*. The Impact of Preoperative Patient Education on Postoperative Pain, Opioid Use, and Psychological Outcomes: A Narrative Review. *Can J Pain*. 2023 Nov 28;7(2):2266751
