# Supplementary material for: Adaptation of Enhanced Recovery After Surgery Protocol for Elective Gastrointestinal and Hepatopancreaticobiliary Surgeries for Tertiary Hospitals in Ethiopia: A Modified Delphi Study
Source: World J Surg. 2026 Mar 2;50(4):860–73. doi: 10.1002/wjs.70274 (PMC13070447; doi:10.1002/wjs.70274)
Supplement: Supplementary file 3 — Supporting Information S3 [file WJS-50-860-s001.docx]

# Appendix 3: Additional Surgical Procedures Proposed for ERAS Protocol Inclusion

| Specialty / Domain | \|  \| \| --- \|  \| Proposed Procedure \| \| --- \| | Notes / Rationale |
| --- | --- | --- | --- | --- |
| Hepatopancreatobiliary (HPB) | - Pancreatic drainage procedures (e.g., for pseudocyst, chronic pancreatitis) - Distal pancreatectomy - Pancreas-preserving duodenal resections - Biliary bypass and reconstruction - Duodenal resections | Expand HPB scope; clarify terminology for pancreatic resections; address complex drainage and reconstruction needs |
| Colorectal Surgery | - Functional colorectal procedures - Perianal surgeries (rectopexy, sphincterotomy, rectal prolapse repair, stoma reversal, hemorrhoid surgery) | Reflects common daily colorectal practice and functional disorder management |
| Upper Gastrointestinal Surgery | - Total gastrectomy - Vagotomy with drainage procedures - Gastrointestinal bypass surgeries | Addresses complex upper GI conditions requiring reconstruction or bypass |
| Surgical Oncology | - Retroperitoneal tumor excision - Mesenteric mass excision | Inclusion of advanced abdominal oncologic procedures |
| General Surgical Principles | - Thoracic and esophageal surgeries (selective ERAS element application) | Certain ERAS components, especially pre- and postoperative care, remain beneficial even if all elements are not feasible |
| Broad Recommendation | - Any surgical procedure, with selective application of ERAS elements as appropriate | Acknowledges universal applicability of ERAS principles with procedure-specific adjustments |
